# Cardiovascular Manifestations of Mitochondrial Disease

**DOI:** 10.3390/biology8020034

**Published:** 2019-05-11

**Authors:** Jason Duran, Armando Martinez, Eric Adler

**Affiliations:** Department of Cardiology, University of California San Diego Medical Center, La Jolla, CA 92037, USA; jaduran@ucsd.edu (J.D.); arm021@ucsd.edu (A.M.)

**Keywords:** mitochondrial, genetic mutations, cardiovascular disease, heart failure, cardiomyopathy

## Abstract

Genetic mitochondrial cardiomyopathies are uncommon causes of heart failure that may not be seen by most physicians. However, the prevalence of mitochondrial DNA mutations and somatic mutations affecting mitochondrial function are more common than previously thought. In this review, the pathogenesis of genetic mitochondrial disorders causing cardiovascular disease is reviewed. Treatment options are presently limited to mostly symptomatic support, but preclinical research is starting to reveal novel approaches that may lead to better and more targeted therapies in the future. With better understanding and clinician education, we hope to improve clinician recognition and diagnosis of these rare disorders in order to improve ongoing care of patients with these diseases and advance research towards discovering new therapeutic strategies to help treat these diseases.

## 1. Introduction

Mitochondrial diseases affect nearly 1 in 5000–10,000 births [1,2,3] and genetic mutations in mitochondrial DNA (mtDNA) are even more commonly found, with studies on umbilical cord blood reporting mutations in as many as 1 in 200 samples [3,4]. Although relatively common, mitochondrial diseases have a wide array of clinical presentations and their dysfunction affects a wide variety of organs and tissues [5] including the heart [6]. As such the diagnosis of a mitochondrial disorder can be complex and easily overlooked. Thus, physician exposure to mitochondrial disorders is quite limited despite its relatively common incidence.

Normal mitochondria serve to supply energy in the form of adenosine triphosphate (ATP), generate and regulate radical oxygen species (ROS), buffer cytosolic calcium ions, and regulate cellular apoptosis via the mitochondrial permeability pore complex [1]. Mitochondria are more richly expressed in tissues with high energy demand, including the heart, brain, skeletal muscle and endocrine system, and their dysfunction will disproportionately affect these systems [7]. Tissues that are more metabolically active will typically have greater vulnerability to defects in mtDNA [8] and they will be affected earlier and more severely than less metabolic tissues [9]. Cardiomyopathy is common and usually increases mortality, with one study reporting that 17% of infants and children hospitalized with an array of mitochondrial diseases had cardiac manifestations, and patients with cardiomyopathy had markedly increased mortality (71%) compared to those without a cardiac phenotype (26%) [10]. Mitochondrial mutations can have variable expression in tissues, and cells can exhibit heteroplasmy in which a single cell can have a mixed population mitochondria with either wild-type or mutant-type mtDNA [8].

The variability of expression of a mutation to mtDNA coupled with the wide variety of symptoms and characteristics of each disorder can make these diseases difficult to identify and diagnose. The diagnostic gold standard of mitochondrial disorders continues to be muscle biopsy, however this is only achieved if physicians are aware of mitochondrial disorders and thinking about them in their differential when diagnosing a patient. In this review, we discuss the pathophysiology of mitochondrial disorders and seek to provide a comprehensive clinical resource on mitochondrial cardiomyopathies and their treatment options. We begin by discussing mitochondrial dysfunction in normal aging, followed by a review of primary (congenital) and secondary mitochondrial disorders with cardiovascular phenotypes. We seek to improve physician recognition and identification of patients with mitochondrial disorders so that we can improve future recognition, diagnosis and treatments of these diseases.

## 2. Mitochondrial Dysfunction in Normal Aging

The heart is one of the most metabolically active tissues in the human body, relying heavily on mitochondrial ATP production to maintain the high energy demand of individually contracting cardiac myocytes. Cardiac aging, or senescence, is coupled with a decline in mitochondrial function, increased production of reactive oxygen species (ROS) and suppressed mitophagy [11,12]. The mitochondrial free radical theory of aging supposes that, with time, free radicals produced as unwanted byproducts of normal mitochondrial metabolism from the respiratory chain on the inner mitochondrial membrane build up in the cell where they are converted to hydrogen peroxide by superoxide dismutase. These hydrogen peroxides are then converted by the Fenton reaction to damaging free radicals that are toxic to nearly all molecules in the cell [12,13]. On the microscopic level, it has been well-established that cells progressively accumulate damage to peptides and lipid molecules with time [14].

In addition to damaging proteins and lipids, free radicals also produces mutations in mitochondrial DNA. Over time, and especially in highly metabolic cells like cardiomyocytes that have high cellular concentrations of mitochondria, ROS-induced molecular damage leads to progressive accumulation of mitochondrial DNA mutations [15,16,17]. This ultimately leads to increasingly greater mitochondrial dysfunction, and, in damaged cells, dysfunctional mitochondria divide and produce more dysfunctional mitochondria that contain greater number of mtDNA mutations [11,17]. Over time, this leads to progressive cellular dysfunction at the organellar level. In metabolically active and mitochondria-rich cells like cardiomyocytes, the cumulation of damaged peptides, lipids and organelles is more pronounced and has a more pronounced contribution to cellular dysfunction and aging [11].

Maintaining protein homeostasis is also central to the normal function of any cell. When peptides get damaged or degraded, the ability to continue normal cellular function relies on the ability to break down and dispose of damage proteins. The cell must also then replace these damage peptides with normally functioning new proteins [11]. The limited ability to remove damaged proteins or replace them can be damaging to normal cell function [18]. Experimental mechanisms that enhance protein homeostasis include reducing insulin-like growth factor-1 (IGF-1) signaling [19,20], caloric restriction or inhibiting mammalian target of rapamycin (mTOR) signaling with rapamycin treatment [21,22]. As the cellular proteome ages, damaged proteins start to build up, protein degradation slows, and cells progressively accumulate more damaged molecules and become progressively more dysfunctional. This is consistent with experimental results that have demonstrated increased levels of protein ubiquitination in the aging heart, [21] but without increased rates of protein turnover [11,21]. On the cellular level, these microscopic changes lead to changes on the organellar and cellular level, with increasingly dysfunctional autophagy with age.

## 3. Primary Mitochondrial Disorders

### 3.1. Mitochondrial Encephalopathy with Lactic Acidosis and Stroke-Like Episodes (MELAS)

First described by Pavlakis et al. in 1984, [23] MELAS has a reported prevalence of 0.18 per 100,000 [24]. Typically presenting in childhood, with 76–80% presenting before 20 years old, the disease rapidly progresses after presentation [25,26]. It has a wide-ranging phenotype that includes stroke-like symptoms (weakness, aphasia, vision loss), encephalopathy (manifesting often as seizures or dementia), lactic acidosis and myopathy [24,26,27]. Additional symptoms include coma, vomiting, fever, headaches, ataxia, external opthalmoplegia, diabetes, hearing impairment, developmental delay, and short stature (Table 1) [26,27].

Cardiac dysfunction has been reported to occur in approximately 30–32% of cases, [24,28] with both hypertrophic and dilated cardiomyopathies having been reported [29]. Conduction abnormalities have also been reported, most notably Wolff–Parkinson–White syndrome [26,30]. The most common genetic mutation involved is A3243G, which has been reported in up to 80% of MELAS presentations [25]. Other mutations that have been identified include T3271C, A3252G, T9957C, 14787del4, G14453A, A13084T, A13045C, A12770G, A11084G, T3949C, G3946A, G3697A, G3376A, T3308C, A13514G, G13513A, G3697, and it is likely several others have yet to be identified (Table 2) [31,32].

Studies have shown treatment with nitric oxide (NO) precursors increase NO production and reportedly reduce stroke-like symptoms [33,34]. One study found plasma lactate decreased significantly after citrulline supplementation [35]. Though no studies have looked directly at the effects of NO precursor therapy on cardiac manifestations, some have theorized they may work by increasing NO production and decreasing cellular damage in cardiac tissue [36]. One case report found improvement in seizure control and reduction in stroke-like episodes after initiation of a ketogenic diet [37]. Reports have shown possible benefits from creatine, CoQ10, and lipoic acid therapy including improvement in symptoms of body composition, strength, and lactate level [38].

### 3.2. Leigh Syndrome

Leigh Syndrome (LS), or subacute necrotizing encephalomyelopathy, was first described by Dr. Denis Leigh, a British neuropsychiatrist, in 1951 [39]. It is a mitochondrial disorder that causes symmetric necrotic lesions in subcortical areas of the brain, particularly the brain stem and basal ganglia. Prevalence is thought to be between 1 per 32,000 and 1 per 40,000 [40,41]. Symptom onset occurs early in life, with initial presentation in infancy or adolescence, and with a majority of presentations before age 1 and 81% by age 2 [42,43]. Initial presentation is often some combination of signs and symptoms of progressive encephalopathy with cognitive and behavioral dysfunction, seizures, hypotonia, oculomotor abnormalities, ataxia, and respiratory dysfunction (Table 1) [40,42,44].

Patients often present with late-stage disease, with 39% of patients dying by the age of 21 years, at a median age of 2.4 years [42,43]. Atypical and slowly progressing presentations have also been described with more rare mutations. Lactate levels are often elevated and diagnosis is usually confirmed by radiologic findings of characteristic bilateral symmetric subcortical hypodensities on computed tomography (CT) or hyperintense signal abnormalities on T2-weighted magnetic resonance imaging (MRI) [42,45,46]. Cardiac manifestations can occur in up to 18–21% of cases, including cardiomyopathy, pericardial effusion, and conduction abnormalities [42,43,47,48]. Of those, hypertrophic cardiomyopathy was found in 50% of patients with cardiac manifestations (Table 1) [43]. One particular study associated the G13513A mutation with development of Wolff–Parkinson–White and hypertrophic cardiomyopathy [48]. One of the major identified causes of LS is Cytochrome c oxidase (COX) deficiency secondary to mutations in the SURF1 gene [49,50]. Over 35 other gene mutations have been identified in association with LS, some of the more studied mutations include 312del10/insAT, T8993C, T8993G, C688T, 772delCC, 751C>T, 845delCT, 868insT, G385A, G618C, T751C, A8344G, A3243G, G13513A, and C1177A (Table 2) [49,50,51,52,53,54].

As with other mitochondrial disorders, treatment options for LS are limited. One case series showed improvement in life-expectancy with Thiamine therapy in patients whose LS was secondary to a SLC19A3 mutation [31]. High dose biotin should be administered to every patient with suspected LS, given similarities with biotin-responsive basal ganglia disease. Some case reports have shown positive results for treating with Coenxyme Q10.

### 3.3. Myoclonic Epilepsy with Ragged Red Fibers (MERRF)

In 1921 Dr. Ramsey Hunt, an American neurologist, first published in the journal *Brain* a syndrome afflicting 6 patients that resembled Freidrich’s ataxia which he described as “dyssynergia cereballaris myoclonica.” It was not until 1973 that Tsairis et al. discovered mitochondrial abnormalities in a family with this disorder, and not until 1980 until Dr. Fukuhara discovered these symptoms associated with ragged red muscle fibers [55]. Finally in 1990 Schoffner et al. first identified a mitochondrial mutation associated with this disease [56]. Myoclonic epilepsy with ragged red fibers (MERRF) has a heterogeneous phenotype that most commonly includes myoclonus, lactic acidosis, cerebellar ataxia, muscle weakness, and ragged red fibers on muscle biopsy [55,57]. Less common symptoms include generalized seizures, short stature, hearing impairment, peripheral neuropathy, headaches, multiple lipomas, vomiting, cognitive dysfunction, and dementia [55,57,58]. Age of onset is typically the second decade of life, and the time of disease progression ranges from 2 to 15 years with a mean time of 8.5 years [58,59]. Reports of the exact incidence of MERRF vary widely, but according to the National Organization for Rare Disorders, the disease occurs in less than 0.9/100,000 patients. Patients often report frequent myoclonic seizures with rare episodes of generalized tonic–clonic seizures, often only partially controlled by anti-epileptic drugs [58]. Reported cardiac abnormalities include Wolff Parkinson White (WPW), supraventricular tachycardia (SVT), right bundle branch block (RBBB), and both dilated and hypertrophic cardiomyopathy (Table 1) [55,59,60,61].

The A8344G mutation is the most commonly identified mutation in patients with MERRF, occurring in 83–90% of cases [57,58]. One case series found cardiac abnormalities in 53% of MERRF patients with this mutation [61]. T8356C has been reported in one patient to be associated with MERRF (Table 2) [62]. G8363A was reported in a case series of 9 patients with clinic presentations fitting MERRF and 4/9 patients were found to cardiac abnormalities [60]. This mutation involves coding for the same tRNA gene as other mutations associated with MERRF. Symptomatic treatment of MERRF is focused on symptomatic management of myoclonus and epilepsy with antiepileptic drugs [58].

### 3.4. Maternally Inherited Diabetes and Deafness (MIDD)

Maternally inherited diabetes and deafness (MIDD), is a mitochondrial disease first characterized in 1992 [63]. Approximately 0.5–2.8% of all diabetic patients have MIDD, with incidences varying by ethnic origin [64,65]. As the name suggests the defining clinical features are diabetes (both type 1 and 2) and bilateral neurosensory hearing loss, each one being equally likely to be the presenting symptom [66]. Typically diabetes develops at a relatively younger age (with half of all patients presenting before age 35), often in a patient with a low or normal BMI [65,66,67,68]. Majority of MIDD patients are non-insulin dependent at presentation however approximately half will have relatively rapid progression to inulin dependence within the first several years of diagnosis [65,66,69,70]. Studies have found macular pattern dystrophy in up to 85% of patients [66,71]. Other less frequent findings include myopathy, neuropsychiatric symptoms, short stature and renal disease [66,69]. Brain atrophy, ptosis, constipation, diarrhea, and pseudo-obstruction have also been reported [72,73,74]. Several case reports have shown a possible association with histories of spontaneous abortion, preterm pregnancy, and placenta accrete (Table 1) [74,75].

Other studies have found left ventricular hypertrophy in up to 55% of patients and cardiomyopathy in up to 15–30% [66,67,76,77]. One study found myocyte hypertrophy and vacuolization, an increased number of mitochondria and evidence of large mitochondria on endomyocardial biopsy [77]. Conduction disorders including WPW, sick sinus syndrome and atrial fibrillation have been found in up to 27% of cases (Table 1) [66,67,68,69]. Another study has shown diabetics with the A3243G mutation had more impairment in autonomic nervous function when compared to other diabetics [78]. The mitochondrial mutation A3243G causes a majority of the cases of MIDD, however other mutations have been identified in rare cases, including point mutations at 568 and 8281 (Table 2) [63,64,79].

Treatment of MIDD is primarily symptomatic. MIDD diabetes is managed similarly to other forms with the exception of avoidance of metformin and low threshold for insulin initiation [65,80]. Hearing impairment is closely followed and patients often benefit from cochlear implantation [65,81]. Case reports have shown treatment with Coenzyme Q10 appeared to improve left ventricular function in patients with MIDD [77,82]. One open trial study conducted on 28 patients in Japan found that treatment with CoQ10 reduced progression of insulin secretion defects, exercise intolerance, and hearing loss [83]. Visual symptoms, like acuity loss, are rare in MIDD [71]. 

### 3.5. Neuropathy, Ataxia and Retinitis Pigmentosa (NARP)

Classically the syndrome of neuropathy, ataxia, and retinitis pigmentosa (NARP) has been described as a combination of deafness, a myoclonic epilepsy, muscle weakness, retinal pigmentosa, and ataxia [84]. The disease was first described by Fryer et al. in 1994 in a family with seven children who presented with a heterogenous phenotype of Leigh’s syndrome [85]. Further investigations however have shown NARP to be phenotypically variable ranging from mild or late-onset symptoms like retinal dystrophy to a more severe multisystemic phenotype that can include developmental delay, dementia, seizures, muscle weakness, sensory neuropathy, ataxia, and retinal pigmentosa and with typical age of onset in the first year of life [84,86]. NARP is estimated to effect about 1/12,000–40,000 according to the National Organization of Rare Disorders and the National Institutes of Health. Several studies have shown a correlation between mutation load and severity of symptoms, especially in the more common mutations at 8993 [87]. Individuals with 80% or more are more likely to have the classic presentation, those below 70% have milder symptoms, and below 50% is often asymptomatic or late onset [86,88,89,90]. Individuals with >90% mutation load appear to present with Leigh syndrome, indicating clinical presentations are on a NARP/Leigh spectrum (Table 1) [91,92].

Further studies have shown that factors other than mutation load affect a patient’s phenotype. Proposed variables for further investigation include mtDNA background, environmental, autosomal, tissue-specific factors, and nuclear modifier genes likely play a role in determining clinical picture and disease severity [93,94,95]. One case report followed a patient with NARP who developed peripartium dilated cardiomyopathy, ventricular pre-excitation, and non-sustained ventricular tachycardia during 4 years of follow up [96]. However this patient had a A8344G, which has been associated with MERRF and not NARP. Though the 8993 mutation has been linked to cases of hypertrophic cardiomyopathy, cases have been in patients with the more severe Leigh syndrome phenotype [97]. More studies focused on long term follow up to determine late sequelae of NARP are needed to determine cardiac outcomes. NARP is caused by mutations in the MT-ATP6 gene, most commonly T8993G and occasionally T8993C [84,86,88]. Other reported mutations include G8839C, G8989C, 8618insT, T9185C, and a 2-bp microdeletion 9127e9128 del AT [89,91,98,99,100]. Other mutations in the MT-ATP6 gene have been documented but have been observed to cause the more severe presentations seen in Leigh syndrome [95,101]. A recent study demonstrated the promise of using mitochondrially targeted obligate heterodimeric zinc finger nucleases to target mitochondrial DNA with the NARP T8993G mutation in cell models [102]. Otherwise treatment has primarily been on symptom improvement (Table 2).

### 3.6. GRACILE

First described in 1998, GRACILE syndrome is a severe mitochondrial disease named after the significant clinical features: growth restriction, aminoaciduria, cholestasis, iron overload, and early death [103]. Incidence is approximated to be at least 1/47,000 based on a study conducted in Finland, however its global prevalence may actually be lower as Finland reports the highest number of cases of GRACILE worldwide [104]. Onset begins with growth restriction at the end of the first trimester, with patient’s average birth weight at standard deviations below predicted normal [105]. A study of 29 GRACILE cases found a median lactate of 12.8 mmol/L, pH of 7.00, and all patients had elevated transaminases [106]. The liver develops macrovesicular steatosis, cholestasis with iron accumulation, rapidly progressive fibrosis and cirrhosis [107]. Lactic acidosis is present and often refractory to treatment, further contributing to failure to thrive. Cause of death appears to occur due to energy depletion, with approximately half of all patients dying within the first two weeks of life and the remainder die by 4 months of life [104,106,107]. One patient was found to have prolonged QT [105]. Autopsy-derived myocardial tissue samples showed higher respiratory chain enzyme activity when compared to autopsy controls [104]. Another study on isolated complex III from GRACILE patients found reduced levels and activity of complex III in myocardial tissues, however no cardiac dysfunction or histopathological abnormalities were found (Table 1) [108].

GRACILE is caused by the homozygous point mutation A232G within the BCS1L gene [104]. BCS1L is a mitochondrial chaperone protein that guides the assembly of complex III of the mitochondrial respiratory chain [104]. It is the most common and severe BCS1L disorder, however multiple mutations within the BCS1L gene have been identified and are linked to a variety of phenotypes [109,110]. Identified exclusively in Finland and included as part of the Finnish disease heritage, it is postulated to have accumulated in the Finnish population through founder effect and genetic drift [105,106]. Patients with the homozygous A232G have shown a strong consistency in the resulting phenotype [111]. BCS1L deficiency did not result in decreased complex III activity in tissues from Finnish GRACILE patients, but patients with BCSL1 deficiency did demonstrate reduced Rieske FeS levels [104]. While the pathophysiology is still largely unknown, a recent study showed tissue specific deficiencies in BCS1L and Rieske FeS proteins in the liver, kidney, and heart tissues were associated with decreased levels of assembled complex III (Table 2) [108]. This pathology was identified only in kidney and liver samples, so despite cardiac tissue also having decreased levels/activity of complex III, there appears to still be adequate respiratory chain enzyme activity [107,108]. Given rapid mortality in these patients, treatment is often symptomatic or palliative [112]. Different methods have been implemented to attempt to correct acidosis without clear success [112]. One study in a mouse model of GRACILE syndrome found decreased survival in mice fed a high carbohydrate diet, despite improved levels of several amino acids and urea cycle intermediates [113].

### 3.7. Mitochondrial Neurogastrointestinal Encephalopathy (MNGIE)

Mitochondrial neurogastrointestinal encephalopathy (MNGIE) is an autosomal recessive disease of multiple organ systems characterized by chronic intestinal dysmotility, leukoencephalopathy, failure to thrive, ptosis, ophthalmoparesis, and peripheral neuropathy [114,115,116]. Gastrointestinal (GI) symptoms are the most prominent manifestation and include recurrent nausea, vomiting, diarrhea, abdominal pain, gastroparesis, and intestinal pseudo-obstruction with dymotility [117,118]. Brain leukoencephalopathy is present in nearly all cases, but is largely asymptomatic [119]. Brain MRI typically shows symmetric T2 hyperintensities of cerebral white matter [119]. Reported in only ~100 cases, patients classically present between the second and fifth decades of life and die in early adulthood as symptoms progress, with mean age at onset around 18 years of age and mean age at death of about 35 years [117,120,121]. One study found abnormal electrocardiogram (ECG) findings, with 16% of patients in one study demonstrating left ventricular hypertrophy (LVH), and there are single case reports of prolonged QT, cardiac arrest, and SVT (Table 1) [120,121].

MNGIE is caused by loss of function mutations to the thymidine phosphorylase (TP) gene located on chromosome 22q13.32-qter (Table 2) [116]. TP catabolizes thymidine to thymine and loss of enzymatic activity causes accumulation of thymidine and deoxyuridine creating an imbalance in reservoir of cellular nucleosides and nucleotides [117]. This results in impaired mitochondrial DNA replication that can lead to further deletions and mutations [122]. TP is expressed in the GI system, brain, peripheral nerves, spleen and bladder, [120] a distribution pattern that accounts for most clinical findings. Despite low expression in muscles, muscle biopsies show mitochondrial dysfunction with abnormal mtDNA, COX deficiency, and ragged-red fibers [120,121,123]. Given the variability of symptom severity and progression without clear correlation between genotype and phenotype, it is hypothesized that environmental factors and genetic modifiers may play a role in determining a patient’s clinical course [121].

Relative to other mitochondrial diseases, significant advancements have been made towards the treatment of MNGIE, but early diagnosis and treatment is crucial to avoid disease progression and accumulation of mutations [124]. Given significant GI manifestations and malnutrition, preventative treatment with nutritional therapy is vital but challenging [125]. Allogeneic hematopoietic stem cell transplant has shown to be a viable treatment option [124,126,127,128]. Stem cell transplantation can restore TP activity and normalizes the pool of nucleotide and nucleoside [126,127]. Despite these molecular improvements, there remains a high complication rate in transplanted patients, with one study showing only 37.5% of patients alive at time of study follow up [128]. Other treatments that have been studied include platelet infusions, peritoneal dialysis, hemodialysis, and carrier erythrocyte entrapped recombinant TP all of which were shown to partially and temporarily restore TP activity levels to varying degrees [129,130,131,132]. Liver transplantation has succeeded in producing lasting restoration of TP activity and symptom improvement in a single case report, though further investigation is needed [133]. Gene therapy-based models for restoration of normal TP enzyme expression has been studied in murine models with promising initial findings [134].

### 3.8. Barth Syndrome

Barth syndrome, first described in 1983 by Dutch neurologist Dr. Peter Barth, [135] is an X-linked mitochondrial disorder causing congenital dilated cardiomyopathy, skeletal myopathy (predominantly proximal), neutropenia (can be intermittent), and growth retardation in young male infants [135,136,137,138]. Prevalence has been estimated to be approximately 1:300,000–400,000 live births, though this is likely an underestimation due to relatively high numbers of spontaneous abortions and still births of male fetuses observed in known Barth syndrome families [139,140]. Disease symptoms typically present within the first year of life with failure to thrive and cardiac abnormalities with or without severe infections, and some studies have even shown cardiac abnormalities in utero [141,142,143]. Mortality is highest within the first four years of life with most deaths due to either cardiac failure secondary to dilated cardiomyopathy or severe sepsis in the setting of neutropenia [136,137,143]. Reported overall survival rate is 49% at age 5, but this has been steadily increasing in recent years, improving from 20% in patients born before 2000 to 70% in patients born after 2000, likely due to increasing awareness leading to earlier diagnosis and treatment [143]. Despite the high mortality rates in childhood, patients can live into their late 40s (Table 1) [141].

As many as 91–94% of patients develop structural cardiac manifestations including dilated cardiomyopathy, endocardial fibroelastosis, or left-ventricular non-compaction (hypertrabeculation) [137,143,144,145,146,147,148]. Conduction abnormalities are also common and include prolonged QT, WPW, SVT, and ventricular tachyarrhythmias [143,146,147]. Neutropenia occurs in 70–84% of patients with variable severity and can be persistent or intermittent [148,149]. Elevations in urine 3-methylglutaconic acid and abnormally low cholesterol levels are less common but still present in a significant percentage of patients (Table 1) [143,147,150].

Barth syndrome is an X-lined recessive disorder caused by mutations in the G4.5 gene (TAZ gene) on Xq28, which encodes a highly conserved ascyltransferase (Table 2) [138,151,152]. The TAZ gene encodes an enzyme important in remodeling of cardiolipin [153,154]. Cardiolipin is exclusive to the mitochondrial membrane and plays a key role in proper structure and function of the mitochondria [153,155]. Loss of this enzyme leads to a relative excess of monolysocardiolipin. Measurement of monolysocardilipin/cardiolipin ratio is an important diagnostic test with high sensitivity and specificity for Barth syndrome [156,157]. Though there is currently no curative treatment for Barth syndrome, early recognition and diagnosis can allow for aggressive and prophylactic management [140,158]. Treatment entails typical heart-failure medication regimens and antibiotics during periods of neutropenia or infection [158]. Despite medical management, approximately 12% of patients require cardiac transplantation, with mean age 3.8 years at time of transplant [148].

### 3.9. Leber’s Hereditary Optic Neuropathy (LHON)

This disorder was first described by Dr. Albrecht von Graefe in 1858, but was named after Dr. Theodore Leber who published a case series in 1871 of 15 patients from 4 families afflicted with LHON [159]. LHON classically presents as acute or subacute, painless vision loss typically in young adulthood although symptom onset varies widely and has been reported as early as 2 and as late as 87 years [160,161,162,163]. One study showed 11.5% of cases occurring before age 10 [161]. Vision loss typically starts in one eye starting with central vision loss and then progresses to the second eye often days to months later [162,163]. Vision loss is due to degeneration of retinal ganglion cells [164]. Other non-visual, neurologic clinical findings have been reported including: dystonia, peripheral neuropathy, resting tremor, wide spread multiple sclerosis-like white matter lesions and Leigh-like encephalopathy [165,166,167,168,169,170,171,172]. European studies have estimated the prevalence to be between 1:31,000–50,000 with a 80–90% of cases occurring in males [173,174,175]. Case reports have found left ventricular hypertrabeculation in patients with LHON [176,177]. Another study showed a statistically significant longer QTc in LHON subjects from one American family when compared to controls [178,179]. Other cardiac associations reported include WPW (~9% of LHON subjects) and myocardial thickening (Table 1) [177,179,180,181].

Approximately 90% cases are caused by one of three mtDNA mutations: G11778A (ND4 gene), G3460A (ND1 gene), and the T14484C (NG6 gene) all of which cause dysfunction in complex I of the mitochondrial respiratory chain (Table 2) [182,183,184,185]. Other mutations have been reported in rare cases [186,187,188,189]. A majority of mutation carriers do not develop clinical LHON, with 50% of male and 10% of female carriers affected [190]. The reason for this pattern is unclear, but several hypotheses of additional determining factors have emerged including varying levels of oxidative stress, environmental factors, mtDNA haplogroups, and hormones [191,192,193,194,195]. Earlier onset of symptoms and the 14484 mutation have relatively higher rates of spontaneous recovery of vision and are associated with better prognosis [196,197].

Several promising therapies are under investigation. A small, open-label, prospective trial looking at drug EPI-743, which targets glutathione production, showed arrest and reversal of disease progression in 4 of 5 subjects [198]. Some case reports, one double-blinded RCT, and one retrospective study have pointed to beneficial effects on vision with a strong antioxidant, idebenone [199,200,201,202]. Given the observed gender bias in disease penetration, studies have begun to investigate the role of estrogen-like molecules on LHON cell-lines with promising preliminary results [203]. A handful of gene-therapy clinical trials are currently underway after promising findings in pre-clinical trials with adeno-associated viral vectors, [204,205,206,207,208] but no studies have evaluated the efficacies of these treatment on cardiac manifestations of this disease.

### 3.10. Pearson Syndrome

Pearson syndrome (PS), first described by pediatric hematologist-oncologist Dr. Howard Pearson in 1979, is a rare disease affecting the liver, kidney, pancreas, bone marrow and CNS, and more rarely can present with cardiovascular findings. This disease presents initially with transfusion-dependent anemia that may improve, but affected patients develop increasingly severe infections [209]. Incidence was estimated to be roughly 1/1,000,000 in a case series of 11 Italian children, [209] and mortality was high with most infants dying by 3 years of age, [210] although later studies have reported survival of one patient to 6.6 years of age [209]. Multiple cases have been reported of both phenotypic and genetic transformation of the disease from PS to Leigh syndrome (with development of dysphagia, ataxia, peripheral neuropathy and ophthalmoparesis) [211,212] or Kearn’s sayre syndrome (with development of progressive external ophtalmoplegia, retinopathy and ataxia) [212,213,214,215]. Cardiac abnormalities, while not hallmark symptoms of the disease, have been reported including increased ventricular wall thickness, depolarization abnormalities and prolonged QT. Mortality likely results from failure of other organ symptoms, as most patients die before any severe cardiac abnormalities can result (Table 1) [209].

Genetic deletions of mtDNA in this disorder is widely variable, and may account for why PS can transform into other mitochondrial spectrum disorders like Kearns Sayre Syndrome (KSS), progressive external ophthalmoplegia (PEO) or Leigh Syndrome (LS) [214]. In a study of 15 patients diagnosed with PS, large mitochondrial DNA deletions were typical, with 9 patients having 4.9 kb deletions and 6 having deletions ranging from 9 to 14 kb (Table 2) [210]. Treatment of this disease primarily involves treatment of anemia with transfusions, erythropoietin (EPO) or granulocyte colony stimulating factor (GCSF), and replacement of pancreatic enzymes has been reported in patients with exocrine pancreatic insufficiency [209].

### 3.11. Kearns–Sayre Syndrome

Kearns–Sayre syndrome (KSS) is a rare mitochondrial disorder that primarily affects the eyes among many other organs including the heart, was first reported by Dr. Kearns with a case series involving nine patients in 1965, and has more recently been described on the spectrum of PEO and PS [212,213,214,215]. Most patients with KSS develop the hallmark symptom of progressive external ophthalmoplegia which causes ptosis and weakness or paralysis of the muscles of the eye and some patients may also develop retinopathy [216]. Cardiac conduction defects are common, as are ataxia and abnormally high cerebrospinal fluid (CSF) protein levels (Table 1). This disorder is very rare, effecting only 1–3 per 100,000 individuals and are caused by large deletions in mtDNA ranging from 1000 to 10,000 base pairs that results in loss of genes involved in oxidative phosphorylation (Table 2) [216]. For cardiac manifestations, AV block is a common complication, with case reports of cardiac arrest and conduction abnormalities including complete heart block requiring permanent cardiac pacemaker implantation (Table 1) [217,218,219,220]. All patients with KSS or KSS spectrum disorders should be regularly screened with ECGs to monitor for progressive AV block, and there should be low threshold for monitoring in any KSS patient presenting with syncope.

### 3.12. Chronic Progressive External Ophthalmoplegia

First reported by Zeviani et al. in 1989, [221] chronic progressive external opthalmoplegia (PEO) is an inherited mitochondrial disorder characterized by loss of motor function of the muscles of the eye an eyelid [222,223,224]. Other associated symptoms include skeletal and cardiac myopathy and dysphagia (Table 1) [223]. Cardiac arrhythmias may also develop. PEO results from large scale mtDNA deletions can occur as part of a spectrum of other large deletion mitochondrial disorders including Kearns–Sayre syndrome [222,223] and Pearson Syndrome, [225,226,227] as mentioned above. Disease incidence of large mtDNA deletions ranges from 1.2–2.9/100,000 [228,229]. Autosomal dominant PEO [230] has also been reported with mutations reported to 3 nuclear encoded genes mutations have been identified in three nuclear encoded genes: *ANT1*, *C10orf2* and *POLG* (Table 2). As with KSS and other large deletion spectrum disorders, symptomatic supportive care remains the only treatment option [224].

### 3.13. Friederich’s Ataxia

Friederich’s ataxia (FA), first described by German physician Dr. Nikolaus Freiderich in 1863, is a neurodegenerative disease that presents with progressive ataxia and areflexia, and progressive life-threatening cardiomyopathy, and both hypertrophic and dilated cardiomyopathies have been reported [231,232]. FA has an incidence of 1–47:1,000,000 [233] that typically presents in childhood, with a mean life expectancy is 40 years of age [234]. About two-thirds of patients will die from cardiac causes (Table 1) [234,235,236]. FA is an autosomal recessive disease caused by expansion of DNA triplet intron repeat GAA in the frataxin (FXN) gene (Table 2) [233,234,237]. Frataxin is a protein found on the inner mitochondrial membrane and is involved in oxidative phosphorylation, and its deficiency can lead to decreased cellular ATP production and disruption of iron chelation within mitochondria [237]. Treatment relies on antioxidant therapy to reduce buildup of reactive oxygen species and decrease oxidative damage. One novel drug, idebenone, an antioxidant that is similar to coenzyme Q, has been suggested for treatment of FA cardiomyopathy early in disease course based on preclinical studies, [238] and has been shown in randomized blinded trial to reduce measures of cardiac hypertrophy in FA-cardiomyopathy patients [239]. 

## 4. Secondary Mitochondrial Myopathies

### 4.1. Mitochondrial Dysfunction in Ischemia

During myocardial infarction, rupture of an atherosclerotic plaque leads to occlusion of a coronary artery and downstream myocardial ischemia. Endocardial myocytes are the most sensitive to cardiac ischemia, as this segment of the myocardium has the highest imbalance of energy, with cell death starting as early as 20 minutes after occlusion of the infarct related artery [240]. The ischemic insult then moves transmurally from the endocardium to the epicardium. In the era of cardiac catheterization, prompt revascularization has served to rapidly restore blood flow to these damaged tissues, however the consequence of this early restoration of blood flow is reperfusion injury, which produces an additional wave of cell death after the primary insult. The combination of ischemia and reperfusion leaves individual cells in three states of injury: dead cardiomyocytes, reversibly injured cells, and hypoperfused but viable cardiomyocytes [240]. Prompt reperfusion has been successful at saving hypoperfused viable cardiomyocytes, but studies have shown that few reversibly injured cells are rescued after reperfusion [240,241]. Additionally, reperfusion causes a second wave of cell death from altered metabolism of cells damaged by ischemia-reperfusion injury, which results in destruction of mitochondria and release of excessive ROS into damaged cells [240,241,242]. In animal studies, as many as 38% of cell death results solely from the reperfusion phase of injury, [242] and reperfusion injury accounts for up to 50% of the final infarct size [241].

Damaged mitochondria can continue to propagate myocyte cell death through activation of programmed cell death pathways. Mitochondrial electron transport chains are damaged during the ischemic phase of injury [243]. This results in excessive production and release of ROSs, dysregulation of calcium handling, and abnormal mitochondrial swelling leads to mitochondrial membrane disruption by the mitochondrial permeability transition pore in the inner mitochondrial membrane, [244,245,246] or via mitochondrial outer membrane permeabilization [246]. Formation of these pore complexes leads to release of toxic mitochondrial ROSs and proteins that activate programmed cell death mechanisms [240,242,243,244,246,247]. Thus, widespread cell death occurs in several stages following coronary artery occlusion, resulting from the ischemic phase, reperfusion phase, and mitochondrial dysfunction phase resulting in cell death both through necrosis and activation of programmed cell death pathways [240,242,244,247].

### 4.2. Mitochondrial Dysfunction in Diabetic Cardiomyopathy

There is growing clinical and preclinical evidence that mitochondrial dysfunction is central to cardiomyopathy observed in diabetic patients [248]. The pathogenesis of mitochondrial dysfunction in diabetes may be similar to that of ischemia-reperfusion, but the exact molecular mechanisms of this dysfunction remain poorly understood [248]. Clinically, diabetic cardiomyopathy was first described over 40 years ago in a case series of four patients with heart failure and diabetes, but with normal coronary arteries [249]. Subsequent studies have confirmed these observations [250,251] and more recent studies estimate the prevalence of diastolic heart failure in as many as 60% of type 2 diabetics [252]. While the exact molecular mechanisms of diabetic cardiomyopathy remains unknown, it is likely a multifactorial process involving disruption of normal respiration via the electron transport chain. This leads to increased ROS production and disrupted fatty acid oxidation.

As outlined by the mitochondrial free radial theory of aging, higher levels of ROS lead to hydroxylation and damage of cellular peptides and lipids [248]. These pathologic changes also result in impaired respiration. Normal hearts primarily generate ATP from mitochondrial fatty acid oxidation (60–70% of ATP generated) and less so from glucose, lactate or other substrates (30–40%) [248,253]. However, in the diabetic hearts, mitochondria are more reliant on fatty acid oxidation and suffer from impaired mitophagy, with greater inability to break down oxidative fatty acids, further perpetuating accumulation of oxygen free radical species. This disrupts mitochondrial calcium handling and myocardial E-C coupling, and elevated levels of cellular ROS can lead to disrupted mitochondrial biogenesis, increased mitochondrial permeability and ultimately increased cell death [248].

## 5. Conclusions

In this review, we seek to improve clinician awareness and recognition of mitochondrial disorders affecting the cardiovascular system. Because of the wide variability of presentations and their relative infrequency in the global population, recognition and diagnosis of these disorders can be challenging. Treatment options for primary mitochondrial diseases are limited and primarily involve support care, although some early studies suggest a role for antioxidant therapy. Future research will be needed to discover novel therapeutic approaches to advance our care of these diseases, including breakthroughs in gene therapy and improved understanding of the genetic expression and penetrance of these mutations. It is our hope that with better recognition of these disorders by clinicians that we can improve diagnosis of these disorders and advance long-term patient care and research into new and more viable treatment options.

## Figures and Tables

**Table 1:** Summary of mitochondrial disorders with cardiac phenotypes.

Disease	Incidence Age at Onset/Death	Primary Phenotype	Cardiac Manifestations	Genetic Mutations	Treatments
MELAS	0.18/100,000 <20 years Rapid progression to death after onset	Stroke-like symptoms Encephalopathy Lactic Acidemia Myopathy	30% of cases: HCM, DCM Conduction abnormalities (WPW)	A324G (80% of cases)	Nitric oxide precursors Citrulline supplementation Ketogenic diet Creatine, CoQ10, lipoic acid therapy
Leigh Syndrome	1/32,000–40,000 <1–2 years Median age of death 2.4 years	Encephalopathy with cognitive and behavioral dysfunction Seizures Hypotonia/Ataxia Oculomotor dysfunction Respiratory dysfunction	20% of cases: HCM Pericardial effusions Conduction abnormalities	Mutations to SURF1 gene G13513A (WPW and HCM)	High dose biotin Thiamine for SLC19A3 CoQ10
MERRF	0.9 or <1/100,000 10–20 years Progression to death within 2–15 years of onset (median 8.4 years)	Myoclonus Lactic acidosis Cerebellar ataxia Muscle weakness Ragged red fibers	DCM HCM Conduction abnormalities (WPW, SVT, RBBB)	A8344G (83–90% of cases and 53% of cases with cardiac involvement)	Treatment of seizures with antiepileptic drugs
MIDD	6/100,000 (~1% of patients with diabetes) <35 years	Diabetes (type I or II) Bilateral neurosensory hearing loss	LVH (55%) HCM (15–30%) Conduction abnormalities (WPW, SSS, Afib)	A3243G	Treatment of diabetes Cochlear implantation CoQ10
NARP	1/12,000–40,000 3-12 months	Neuropathy Ataxia Retinitis Pigmentosa Deafness Myoclonic epilepsy	DCM HCM Conduction abnormalities (WPW)	Point mutations at 8993 MT-ATP6 gene (most commonly T8993G, then T8993C)	Experimental: mitochondrially targeted obligate heterodimeric zinc finger nucleases to target mitochondrial DNA with the NARP T8993G mutation
GRACILE	1/47,000 (in Finland, may be lower worldwide) Onset in utero 50% die within first 4 months of life, remainder die by 4 years	Growth restriction Aminoaciduria Cholestasis Iron overload Early death	Prolonged QT Reduced levels of complex III in myocardial tissues post mortem	homozygous point mutation A232G within the BCS1L gene	Symptomatic/palliative care (usually lethal in first months of life).
MNGIE	* Only 100 cases ever reported Mean age of onset 18 years Mean age of death 35 years	Chronic intestinal dysmotility Leukoencephalopathy Failure to thrive Ptosis Ophthalmoparesis Peripheral neuropathy	LVH Conduction abnormalities (prolonged QT, SVT, sudden cardiac death)	Loss of function mutations to thymidine phosphorylase (TP) gene, chromosome 22q13.32-qter	Allogeneic hematopoietic stem cell transplantation Platelet transfusion Hemodialysis or peritoneal dialysis Experimental gene therapies aimed to restore thymidine phosphorylase activity
Barth Syndrome	1:300,000–400,000 <1 year Most die within first 4 years of life	DCM Skeletal myopathy (proximal) Neutropenia Growth retardation	DCM Cardiac abnormalities in utero Endocardial fibroelastosis LV noncompaction Conduction abnormalities (prolonged WT, SVT, WPW, VT)	G4.5 gene (TAZ gene) on Xq28	Treatment of heart failure Cardiac transplantation
LHON	1/31,000–50,000 2–87 years	Acute/subacute painless vision loss Dystonia Peripheral neuropathy	LV hypertrebeculation Prolonged QT WPW	90% caused by G11778A (ND4 gene), G3460A (ND1 gene), and the T14484C (NG6 gene) which all cause dysfunction in complex I	EPI-743: experimental drug targeting glutathione production Idebenone (antioxidant that can slow vision loss)
Pearson Syndrome	1/1,000,000 Presents in infancy most deaths by 3 years of age	Transfusion-dependent anemia (presenting finding) Severe Infections Liver, Kidney, Pancreas and CNS abnormalities	Increased wall thickness Depolarization abnormalities Prolonged QT	Large deletions ranging from 4.9–14 kb	Treatment of anemia with transfusions, EPO, GCSF Treatment of pancreatic insufficiency with enzyme replacement
Kearns-Sayre Syndrome	1–3/100,000	Progressive external ophthalmoplegia (ptosis, weakness of eye muscles) Retinopathy Ataxia Elevated CSF proteins	Conduction abnormalities (AV block requiring PPM) Cardiac arrest	Large deletions ranging from 1000 to 10,000 base pairs	ECG screening PPM implantation for AV block
CPEO	1–3/100,000	Loss of motor function of the eye and eyelid Spectrum of disorders with PS and KSS	HCM Arrhythmias	Large mtDNA deletions similar to PS or KSS AD: mutations to nuclear-encoded genes ANT1, C10orf2 and POLG	Symptomatic/supportive care
Freiderich’s Ataxia	1–47:1,000,000 Presents in childhood Mean life expectancy 40 years	Progressive ataxia/areflexia Progressive and life-threatening cardiomyopathy (2/3 patients die from cardiovascular causes)	HCM DCM	Expansion of DNA triplet intron repeat GAA in the frataxin (FXN) gene	Idebenone (antioxidant), reduces cardiac hypertrophy

Abbreviations: hypertrophic cardiomyopathy (HCM), dilated cardiomyopathy (DCM), Wolff-Parkinson-White syndrome (WPW), supraventricular tachycardia (SVT), right bundle branch block (RBBB), left ventricular hypertrophy (LVH), sick sinus syndrome (SSS), atrial fibrillation (Afib), ventricular tachyarrhythmias, permanent pacemaker (PPM), autosomal dominant (AD).

**Table 2:** Genetic mutations associated with mitochondrial disorders.

Disease	Mutations
MELAS	**A324G (80% of cases)**, T3271C, A3252G, T9957C, 14787del4, G14453A, A13084T, A13045C, A12770G, A11084G, T3949C, G3946A, G3697A, G3376A, T3308C, A13514G, G13513A, G3697
Leigh Syndrome	Mutations to **SURF1** gene, G13513A (WPW and HCM), 312del10/insAT, T8993C, T8993G, C688T, 772delCC, 751C>T, 845delCT, 868insT, G385A, G618C, T751C, A8344G, A3243G, G13513A, and C1177A.
MERRF	**A8344G**, T8356C, G8363A
MIDD	**A3243G**, point mutations at 568 and 8281
NARP	Point mutations at **8993**, mutations to **MT-ATP6** gene (most commonly **T8993G**, then **T8993C**), G8839C, G8989C, 8618insT, T9185C, and a 2 bp microdeletion 9127e9128 del AT
GRACILE	Homozygous point mutation A232G within the BCS1L gene
MNGIE	Loss of function mutations to **thymidine phosphorylase (TP)** gene, chromosome 22q13.32-qter
Barth Syndrome	**G4.5 gene** (TAZ gene) on Xq28
LHON	90% caused by **G11778A** (ND4 gene), G3460A (ND1 gene), and the T14484C (NG6 gene) which all cause dysfunction in complex I
Pearson Syndrome	Large deletions ranging from 4.9–14 kb
Kearns-Sayre Syndrome	Large deletions ranging from 1000 to 10,000 base pairs
CPEO	Large mtDNA deletions similar to PS or KSS, AD form: mutations to nuclear-encoded genes *ANT1*, *C10orf2* and *POLG*
Freiderich’s Ataxia	Expansion of DNA **triplet intron repeat GAA** in the **frataxin (FXN) gene**

*Principal mutations of each disorder are **bolded** (that most frequently attributed to each disease).

## References

[1] Meyers D.E., Basha H.I., Koenig M.K. (2013). Cardiac manifestations of mitochondrial disorders. Tex. Heart Inst. J..

[2] Meyers D.E., Basha H.I., Koenig M.K. (2013). Mitochondrial cardiomyopathy: Pathophysiology, diagnosis, and management. Tex. Heart Inst. J..

[3] Chinnery P.F., Elliott H.R., Hudson G., Samuels D.C., Relton C.L. (2012). Epigenetics, epidemiology and mitochondrial DNA diseases. Int. J. Epidemiol..

[4] Elliott H.R., Samuels D.C., Eden J.A., Relton C.L., Chinnery P.F. (2008). Pathogenic mitochondrial DNA mutations are common in the general population. Am. J. Hum. Genet..

[5] Debray F.G., Lambert M., Chevalier I., Robitaille Y., Decarie J.C., Shoubridge E.A., Robinson B.H., Mitchell G.A. (2007). Long-term outcome and clinical spectrum of 73 pediatric patients with mitochondrial diseases. Pediatrics.

[6] Anan R., Nakagawa M., Miyata M., Higuchi I., Nakao S., Suehara M., Osame M., Tanaka H. (1995). Cardiac involvement in mitochondrial diseases. A study on 17 patients with documented mitochondrial DNA defects. Circulation.

[7] Lane N. (2011). Evolution. The costs of breathing. Science.

[8] Wallace D.C. (2007). Why do we still have a maternally inherited mitochondrial DNA? Insights from evolutionary medicine. Annu. Rev. Biochem..

[9] Hirano M., Davidson M., DiMauro S. (2001). Mitochondria and the heart. Curr. Opin. Cardiol..

[10] Holmgren D., Wahlander H., Eriksson B.O., Oldfors A., Holme E., Tulinius M. (2003). Cardiomyopathy in children with mitochondrial disease; clinical course and cardiological findings. Eur. Heart J..

[11] Tocchi A., Quarles E.K., Basisty N., Gitari L., Rabinovitch P.S. (2015). Mitochondrial dysfunction in cardiac aging. Biochim. Biophys. Acta.

[12] Bratic A., Larsson N.G. (2013). The role of mitochondria in aging. J. Clin. Investig..

[13] Stadtman E.R. (1992). Protein oxidation and aging. Science.

[14] Balaban R.S., Nemoto S., Finkel T. (2005). Mitochondria, oxidants, and aging. Cell.

[15] Trifunovic A., Larsson N.G. (2008). Mitochondrial dysfunction as a cause of ageing. J. Intern. Med..

[16] Trounce I., Byrne E., Marzuki S. (1989). Decline in skeletal muscle mitochondrial respiratory chain function: Possible factor in ageing. Lancet.

[17] Kujoth G.C., Hiona A., Pugh T.D., Someya S., Panzer K., Wohlgemuth S.E., Hofer T., Seo A.Y., Sullivan R., Jobling W.A. (2005). Mitochondrial DNA mutations, oxidative stress, and apoptosis in mammalian aging. Science.

[18] Koga H., Kaushik S., Cuervo A.M. (2011). Protein homeostasis and aging: The importance of exquisite quality control. Ageing Res. Rev..

[19] Abbas A., Grant P.J., Kearney M.T. (2008). Role of IGF-1 in glucose regulation and cardiovascular disease. Expert Rev. Cardiovasc. Ther..

[20] Puglielli L. (2008). Aging of the brain, neurotrophin signaling, and Alzheimer’s disease: Is IGF1-R the common culprit?. Neurobiol. Aging.

[21] Dai D.F., Karunadharma P.P., Chiao Y.A., Basisty N., Crispin D., Hsieh E.J., Chen T., Gu H., Djukovic D., Raftery D. (2014). Altered proteome turnover and remodeling by short-term caloric restriction or rapamycin rejuvenate the aging heart. Aging Cell.

[22] Johnson S.C., Rabinovitch P.S., Kaeberlein M. (2013). mTOR is a key modulator of ageing and age-related disease. Nature.

[23] Pavlakis S.G., Phillips P.C., DiMauro S., De Vivo D.C., Rowland L.P. (1984). Mitochondrial myopathy, encephalopathy, lactic acidosis, and strokelike episodes: A distinctive clinical syndrome. Ann. Neurol..

[24] Yatsuga S., Povalko N., Nishioka J., Katayama K., Kakimoto N., Matsuishi T., Kakuma T., Koga Y., Taro Matsuoka for M.S.G.i.J. (2012). MELAS: A nationwide prospective cohort study of 96 patients in Japan. Biochim. Biophys. Acta.

[25] Goto Y., Horai S., Matsuoka T., Koga Y., Nihei K., Kobayashi M., Nonaka I. (1992). Mitochondrial myopathy, encephalopathy, lactic acidosis, and stroke-like episodes (MELAS): A correlative study of the clinical features and mitochondrial DNA mutation. Neurology.

[26] Hirano M., Ricci E., Koenigsberger M.R., Defendini R., Pavlakis S.G., DeVivo D.C., DiMauro S., Rowland L.P. (1992). Melas: An original case and clinical criteria for diagnosis. Neuromuscul. Disord..

[27] Zhang J., Guo J., Fang W., Jun Q., Shi K. (2015). Clinical features of MELAS and its relation with A3243G gene point mutation. Int. J. Clin. Exp. Pathol..

[28] Mancuso M., Orsucci D., Angelini C., Bertini E., Carelli V., Comi G.P., Donati A., Minetti C., Moggio M., Mongini T. (2014). The m.3243A>G mitochondrial DNA mutation and related phenotypes. A matter of gender?. J. Neurol..

[29] Okajima Y., Tanabe Y., Takayanagi M., Aotsuka H. (1998). A follow up study of myocardial involvement in patients with mitochondrial encephalomyopathy, lactic acidosis, and stroke-like episodes (MELAS). Heart.

[30] Sproule D.M., Kaufmann P., Engelstad K., Starc T.J., Hordof A.J., De Vivo D.C. (2007). Wolff-Parkinson-White syndrome in Patients With MELAS. Arch. Neurol..

[31] El-Hattab A.W., Adesina A.M., Jones J., Scaglia F. (2015). MELAS syndrome: Clinical manifestations, pathogenesis, and treatment options. Mol. Genet. Metab..

[32] Lott M.T., Leipzig J.N., Derbeneva O., Xie H.M., Chalkia D., Sarmady M., Procaccio V., Wallace D.C. (2013). mtDNA Variation and Analysis Using Mitomap and Mitomaster. Curr. Protoc. Bioinformat..

[33] El-Hattab A.W., Emrick L.T., Hsu J.W., Chanprasert S., Almannai M., Craigen W.J., Jahoor F., Scaglia F. (2016). Impaired nitric oxide production in children with MELAS syndrome and the effect of arginine and citrulline supplementation. Mol. Genet. Metab..

[34] Ganetzky R.D., Falk M.J. (2018). 8-year retrospective analysis of intravenous arginine therapy for acute metabolic strokes in pediatric mitochondrial disease. Mol. Genet. Metab..

[35] El-Hattab A.W., Emrick L.T., Williamson K.C., Craigen W.J., Scaglia F. (2013). The effect of citrulline and arginine supplementation on lactic acidemia in MELAS syndrome. Meta Gene.

[36] El-Hattab A.W., Zarante A.M., Almannai M., Scaglia F. (2017). Therapies for mitochondrial diseases and current clinical trials. Mol. Genet. Metab..

[37] Steriade C., Andrade D.M., Faghfoury H., Tarnopolsky M.A., Tai P. (2014). Mitochondrial encephalopathy with lactic acidosis and stroke-like episodes (MELAS) may respond to adjunctive ketogenic diet. Pediatr. Neurol..

[38] Rodriguez M.C., MacDonald J.R., Mahoney D.J., Parise G., Beal M.F., Tarnopolsky M.A. (2007). Beneficial effects of creatine, CoQ10, and lipoic acid in mitochondrial disorders. Muscle Nerve.

[39] Leigh D. (1951). Subacute necrotizing encephalomyelopathy in an infant. J. Neurol. Neurosurg. Psychiatry.

[40] Rahman S., Blok R.B., Dahl H.H., Danks D.M., Kirby D.M., Chow C.W., Christodoulou J., Thorburn D.R. (1996). Leigh syndrome: Clinical features and biochemical and DNA abnormalities. Ann. Neurol..

[41] Darin N., Oldfors A., Moslemi A.R., Holme E., Tulinius M. (2001). The incidence of mitochondrial encephalomyopathies in childhood: Clinical features and morphological, biochemical, and DNA abnormalities. Ann. Neurol..

[42] Lee H.F., Tsai C.R., Chi C.S., Lee H.J., Chen C.C. (2009). Leigh syndrome: Clinical and neuroimaging follow-up. Pediatr. Neurol..

[43] Sofou K., De Coo I.F., Isohanni P., Ostergaard E., Naess K., De Meirleir L., Tzoulis C., Uusimaa J., De Angst I.B., Lonnqvist T. (2014). A multicenter study on Leigh syndrome: Disease course and predictors of survival. Orphanet J. Rare Dis..

[44] Huntsman R.J., Sinclair D.B., Bhargava R., Chan A. (2005). Atypical presentations of leigh syndrome: A case series and review. Pediatr. Neurol..

[45] Bonfante E., Koenig M.K., Adejumo R.B., Perinjelil V., Riascos R.F. (2016). The neuroimaging of Leigh syndrome: Case series and review of the literature. Pediatr. Radiol..

[46] Sonam K., Khan N.A., Bindu P.S., Taly A.B., Gayathri N., Bharath M.M., Govindaraju C., Arvinda H.R., Nagappa M., Sinha S. (2014). Clinical and magnetic resonance imaging findings in patients with Leigh syndrome and SURF1 mutations. Brain Dev..

[47] Hadzsiev K., Maasz A., Kisfali P., Kalman E., Gomori E., Pal E., Berenyi E., Komlosi K., Melegh B. (2010). Mitochondrial DNA 11777C>A mutation associated Leigh syndrome: Case report with a review of the previously described pedigrees. Neuromol. Med..

[48] Wang S.B., Weng W.C., Lee N.C., Hwu W.L., Fan P.C., Lee W.T. (2008). Mutation of mitochondrial DNA G13513A presenting with Leigh syndrome, Wolff-Parkinson-White syndrome and cardiomyopathy. Pediatr. Neonatol..

[49] Pequignot M.O., Dey R., Zeviani M., Tiranti V., Godinot C., Poyau A., Sue C., Di Mauro S., Abitbol M., Marsac C. (2001). Mutations in the SURF1 gene associated with Leigh syndrome and cytochrome C oxidase deficiency. Hum. Mutat..

[50] Yang Y.L., Sun F., Zhang Y., Qian N., Yuan Y., Wang Z.X., Qi Y., Xiao J.X., Wang X.Y., Qi Z.Y. (2006). Clinical and laboratory survey of 65 Chinese patients with Leigh syndrome. Chin. Med. J..

[51] Tiranti V., Hoertnagel K., Carrozzo R., Galimberti C., Munaro M., Granatiero M., Zelante L., Gasparini P., Marzella R., Rocchi M. (1998). Mutations of SURF-1 in Leigh disease associated with cytochrome c oxidase deficiency. Am. J. Hum. Genet..

[52] Moslemi A.R., Tulinius M., Darin N., Aman P., Holme E., Oldfors A. (2003). SURF1 gene mutations in three cases with Leigh syndrome and cytochrome c oxidase deficiency. Neurology.

[53] Poyau A., Buchet K., Bouzidi M.F., Zabot M.T., Echenne B., Yao J., Shoubridge E.A., Godinot C. (2000). Missense mutations in SURF1 associated with deficient cytochrome c oxidase assembly in Leigh syndrome patients. Hum. Genet..

[54] Gerards M., Kamps R., van Oevelen J., Boesten I., Jongen E., de Koning B., Scholte H.R., de Angst I., Schoonderwoerd K., Sefiani A. (2013). Exome sequencing reveals a novel Moroccan founder mutation in SLC19A3 as a new cause of early-childhood fatal Leigh syndrome. Brain.

[55] Fukuhara N., Tokiguchi S., Shirakawa K., Tsubaki T. (1980). Myoclonus epilepsy associated with ragged-red fibres (mitochondrial abnormalities ): Disease entity or a syndrome? Light-and electron-microscopic studies of two cases and review of literature. J. Neurol. Sci..

[56] Shoffner J.M., Lott M.T., Lezza A.M., Seibel P., Ballinger S.W., Wallace D.C. (1990). Myoclonic epilepsy and ragged-red fiber disease (MERRF) is associated with a mitochondrial DNA tRNA(Lys) mutation. Cell.

[57] Fang W., Huang C.C., Chu N.S., Lee C.C., Chen R.S., Pang C.Y., Shih K.D., Wei Y.H. (1994). Myoclonic epilepsy with ragged-red fibers (MERRF) syndrome: Report of a Chinese family with mitochondrial DNA point mutation in tRNA(Lys) gene. Muscle Nerve.

[58] Lorenzoni P.J., Scola R.H., Kay C.S., Arndt R.C., Silvado C.E., Werneck L.C. (2011). MERRF: Clinical features, muscle biopsy and molecular genetics in Brazilian patients. Mitochondrion.

[59] Ozawa M., Goto Y., Sakuta R., Tanno Y., Tsuji S., Nonaka I. (1995). The 8,344 mutation in mitochondrial DNA: A comparison between the proportion of mutant DNA and clinico-pathologic findings. Neuromuscul. Disord..

[60] Santorelli F.M., Mak S.C., El-Schahawi M., Casali C., Shanske S., Baram T.Z., Madrid R.E., DiMauro S. (1996). Maternally inherited cardiomyopathy and hearing loss associated with a novel mutation in the mitochondrial tRNA(Lys) gene (G8363A). Am. J. Hum. Genet..

[61] Catteruccia M., Sauchelli D., Della Marca G., Primiano G., Cuccagna C., Bernardo D., Leo M., Camporeale A., Sanna T., Cianfoni A. (2015). “Myo-cardiomyopathy” is commonly associated with the A8344G “MERRF” mutation. J. Neurol..

[62] Silvestri G., Moraes C.T., Shanske S., Oh S.J., DiMauro S. (1992). A new mtDNA mutation in the tRNA(Lys) gene associated with myoclonic epilepsy and ragged-red fibers (MERRF). Am. J. Hum. Genet..

[63] van den Ouweland J.M., Lemkes H.H., Ruitenbeek W., Sandkuijl L.A., de Vijlder M.F., Struyvenberg P.A., van de Kamp J.J., Maassen J.A. (1992). Mutation in mitochondrial tRNA(Leu)(UUR) gene in a large pedigree with maternally transmitted type II diabetes mellitus and deafness. Nat. Genet..

[64] Maassen J.A., Janssen G.M., ‘t Hart L.M. (2005). Molecular mechanisms of mitochondrial diabetes (MIDD). Ann. Med..

[65] Murphy R., Turnbull D.M., Walker M., Hattersley A.T. (2008). Clinical features, diagnosis and management of maternally inherited diabetes and deafness (MIDD) associated with the 3243A>G mitochondrial point mutation. Diabet. Med..

[66] Guillausseau P.J., Massin P., Dubois-LaForgue D., Timsit J., Virally M., Gin H., Bertin E., Blickle J.F., Bouhanick B., Cahen J. (2001). Maternally inherited diabetes and deafness: A multicenter study. Ann. Intern. Med..

[67] Suzuki S., Oka Y., Kadowaki T., Kanatsuka A., Kuzuya T., Kobayashi M., Sanke T., Seino Y., Nanjo K., Society R.C.o.S.T.o.D.M.w.G.M.o.t.J.D. (2003). Clinical features of diabetes mellitus with the mitochondrial DNA 3243 (A-G) mutation in Japanese: Maternal inheritance and mitochondria-related complications. Diabetes Res. Clin. Pract..

[68] Zhu J., Yang P., Liu X., Yan L., Rampersad S., Li F., Li H., Sheng C., Cheng X., Zhang M. (2017). The clinical characteristics of patients with mitochondrial tRNA Leu(UUR)m.3243A > G mutation: Compared with type 1 diabetes and early onset type 2 diabetes. J. Diabetes Complicat..

[69] Guillausseau P.J., Dubois-Laforgue D., Massin P., Laloi-Michelin M., Bellanné-Chantelot C., Gin H., Bertin E., Blickle J.F., Bauduceau B., Bouhanick B. (2004). Heterogeneity of diabetes phenotype in patients with 3243 bp mutation of mitochondrial DNA (Maternally Inherited Diabetes and Deafness or MIDD). Diabetes Metab..

[70] Whittaker R.G., Schaefer A.M., McFarland R., Taylor R.W., Walker M., Turnbull D.M. (2007). Prevalence and progression of diabetes in mitochondrial disease. Diabetologia.

[71] Smith P.R., Bain S.C., Good P.A., Hattersley A.T., Barnett A.H., Gibson J.M., Dodson P.M. (1999). Pigmentary retinal dystrophy and the syndrome of maternally inherited diabetes and deafness caused by the mitochondrial DNA 3243 tRNA(Leu) A to G mutation. Ophthalmology.

[72] Narbonne H., Paquis-Fluckinger V., Valero R., Heyries L., Pellissier J.F., Vialettes B. (2004). Gastrointestinal tract symptoms in Maternally Inherited Diabetes and Deafness (MIDD). Diabetes Metab..

[73] Robberecht K., Decock C., Stevens A., Seneca S., De Bleecker J., Leroy B.P. (2010). Ptosis as an associated finding in maternally inherited diabetes and deafness. Ophthalmic Genet..

[74] Lien L.M., Lee H.C., Wang K.L., Chiu J.C., Chiu H.C., Wei Y.H. (2001). Involvement of nervous system in maternally inherited diabetes and deafness (MIDD) with the A3243G mutation of mitochondrial DNA. Acta Neurol. Scand..

[75] Aggarwal P., Gill-Randall R., Wheatley T., Buchalter M.B., Metcalfe J., Alcolado J.C. (2001). Identification of mtDNA mutation in a pedigree with gestational diabetes, deafness, Wolff-Parkinson-White syndrome and placenta accreta. Hum. Hered..

[76] Majamaa-Voltti K., Peuhkurinen K., Kortelainen M.L., Hassinen I.E., Majamaa K. (2002). Cardiac abnormalities in patients with mitochondrial DNA mutation 3243A>G. BMC Cardiovasc. Disord..

[77] Azevedo O., Vilarinho L., Almeida F., Ferreira F., Guardado J., Ferreira M., Lourenço A., Medeiros R., Almeida J. (2010). Cardiomyopathy and kidney disease in a patient with maternally inherited diabetes and deafness caused by the 3243A>G mutation of mitochondrial DNA. Cardiology.

[78] Momiyama Y., Suzuki Y., Ohtomo M., Atsumi Y., Matsuoka K., Ohsuzu F., Kimura M. (2002). Cardiac autonomic nervous dysfunction in diabetic patients with a mitochondrial DNA mutation: Assessment by heart rate variability. Diabetes Care.

[79] Tabebi M., Charfi N., Kallabi F., Alila-Fersi O., Ben Mahmoud A., Tlili A., Keskes-Ammar L., Kamoun H., Abid M., Mnif M. (2017). Whole mitochondrial genome screening of a family with maternally inherited diabetes and deafness (MIDD) associated with retinopathy: A putative haplotype associated to MIDD and a novel MT-CO2 m.8241T>G mutation. J. Diabetes Complicat..

[80] Maassen J.A., ‘T Hart L.M., Van Essen E., Heine R.J., Nijpels G., Jahangir Tafrechi R.S., Raap A.K., Janssen G.M., Lemkes H.H. (2004). Mitochondrial diabetes: Molecular mechanisms and clinical presentation. Diabetes.

[81] Sinnathuray A.R., Raut V., Awa A., Magee A., Toner J.G. (2003). A review of cochlear implantation in mitochondrial sensorineural hearing loss. Otol. Neurotol..

[82] Salles J.E., Moisés V.A., Almeida D.R., Chacra A.R., Moisés R.S. (2006). Myocardial dysfunction in mitochondrial diabetes treated with Coenzyme Q10. Diabetes Res. Clin. Pract..

[83] Suzuki S., Hinokio Y., Ohtomo M., Hirai M., Hirai A., Chiba M., Kasuga S., Satoh Y., Akai H., Toyota T. (1998). The effects of coenzyme Q10 treatment on maternally inherited diabetes mellitus and deafness, and mitochondrial DNA 3243 (A to G) mutation. Diabetologia.

[84] Holt I.J., Harding A.E., Petty R.K., Morgan-Hughes J.A. (1990). A new mitochondrial disease associated with mitochondrial DNA heteroplasmy. Am. J. Hum. Genet..

[85] Fryer A., Appleton R., Sweeney M.G., Rosenbloom L., Harding A.E. (1994). Mitochondrial DNA 8993 (NARP) mutation presenting with a heterogeneous phenotype including ‘cerebral palsy’. Arch. Dis. Child..

[86] Porto F.B., Mack G., Sterboul M.J., Lewin P., Flament J., Sahel J., Dollfus H. (2001). Isolated late-onset cone-rod dystrophy revealing a familial neurogenic muscle weakness, ataxia, and retinitis pigmentosa syndrome with the T8993G mitochondrial mutation. Am. J. Ophthalmol..

[87] Jonckheere A.I., Smeitink J.A., Rodenburg R.J. (2012). Mitochondrial ATP synthase: Architecture, function and pathology. J. Inherit. Metab. Dis..

[88] White S.L., Collins V.R., Wolfe R., Cleary M.A., Shanske S., DiMauro S., Dahl H.H., Thorburn D.R. (1999). Genetic counseling and prenatal diagnosis for the mitochondrial DNA mutations at nucleotide 8993. Am. J. Hum. Genet..

[89] López-Gallardo E., Solano A., Herrero-Martín M.D., Martínez-Romero I., Castaño-Pérez M.D., Andreu A.L., Herrera A., López-Pérez M.J., Ruiz-Pesini E., Montoya J. (2009). NARP syndrome in a patient harbouring an insertion in the MT-ATP6 gene that results in a truncated protein. J. Med. Genet..

[90] Sciacco M., Prelle A., D’Adda E., Lamperti C., Bordoni A., Rango M., Crimi M., Comi G.P., Bresolin N., Moggio M. (2003). Familial mtDNA T8993C transition causing both the NARP and the MILS phenotype in the same generation. A morphological, genetic and spectroscopic study. J. Neurol..

[91] Childs A.M., Hutchin T., Pysden K., Highet L., Bamford J., Livingston J., Crow Y.J. (2007). Variable phenotype including Leigh syndrome with a 9185T>C mutation in the MTATP6 gene. Neuropediatrics.

[92] Tatuch Y., Christodoulou J., Feigenbaum A., Clarke J.T., Wherret J., Smith C., Rudd N., Petrova-Benedict R., Robinson B.H. (1992). Heteroplasmic mtDNA mutation (T----G) at 8993 can cause Leigh disease when the percentage of abnormal mtDNA is high. Am. J. Hum. Genet..

[93] D’Aurelio M., Vives-Bauza C., Davidson M.M., Manfredi G. (2010). Mitochondrial DNA background modifies the bioenergetics of NARP/MILS ATP6 mutant cells. Hum. Mol. Genet..

[94] Claeys K.G., Abicht A., Häusler M., Kleinle S., Wiesmann M., Schulz J.B., Horvath R., Weis J. (2016). Novel genetic and neuropathological insights in neurogenic muscle weakness, ataxia, and retinitis pigmentosa (NARP). Muscle Nerve.

[95] Kara B., Arıkan M., Maraş H., Abacı N., Cakıris A., Ustek D. (2012). Whole mitochondrial genome analysis of a family with NARP/MILS caused by m.8993T>C mutation in the MT-ATP6 gene. Mol. Genet. Metab..

[96] Limongelli G., Tome-Esteban M., Dejthevaporn C., Rahman S., Hanna M.G., Elliott P.M. (2010). Prevalence and natural history of heart disease in adults with primary mitochondrial respiratory chain disease. Eur. J. Heart Fail..

[97] Pastores G.M., Santorelli F.M., Shanske S., Gelb B.D., Fyfe B., Wolfe D., Willner J.P. (1994). Leigh syndrome and hypertrophic cardiomyopathy in an infant with a mitochondrial DNA point mutation (T8993G). Am. J. Med. Genet..

[98] Duno M., Wibrand F., Baggesen K., Rosenberg T., Kjaer N., Frederiksen A.L. (2013). A novel mitochondrial mutation m.8989G>C associated with neuropathy, ataxia, retinitis pigmentosa—The NARP syndrome. Gene.

[99] Mordel P., Schaeffer S., Dupas Q., Laville M.A., Gérard M., Chapon F., Allouche S. (2017). A 2 bp deletion in the mitochondrial ATP 6 gene responsible for the NARP (neuropathy, ataxia, and retinitis pigmentosa) syndrome. Biochem. Biophys. Res. Commun..

[100] Blanco-Grau A., Bonaventura-Ibars I., Coll-Cantí J., Melià M.J., Martinez R., Martínez-Gallo M., Andreu A.L., Pinós T., García-Arumí E. (2013). Identification and biochemical characterization of the novel mutation m.8839G>C in the mitochondrial ATP6 gene associated with NARP syndrome. Genes Brain Behav..

[101] Moslemi A.R., Darin N., Tulinius M., Oldfors A., Holme E. (2005). Two new mutations in the MTATP6 gene associated with Leigh syndrome. Neuropediatrics.

[102] Gammage P.A., Rorbach J., Vincent A.I., Rebar E.J., Minczuk M. (2014). Mitochondrially targeted ZFNs for selective degradation of pathogenic mitochondrial genomes bearing large-scale deletions or point mutations. EMBO Mol. Med..

[103] Fellman V., Rapola J., Pihko H., Varilo T., Raivio K.O. (1998). Iron-overload disease in infants involving fetal growth retardation, lactic acidosis, liver haemosiderosis, and aminoaciduria. Lancet.

[104] Visapää I., Fellman V., Vesa J., Dasvarma A., Hutton J.L., Kumar V., Payne G.S., Makarow M., Van Coster R., Taylor R.W. (2002). GRACILE syndrome, a lethal metabolic disorder with iron overload, is caused by a point mutation in BCS1L. Am. J. Hum. Genet..

[105] Fellman V. (2002). The GRACILE syndrome, a neonatal lethal metabolic disorder with iron overload. Blood Cells Mol. Dis..

[106] Fellman V., Kotarsky H. (2011). Mitochondrial hepatopathies in the newborn period. Semin. Fetal Neonatal Med..

[107] Rapola J., Heikkilä P., Fellman V. (2002). Pathology of lethal fetal growth retardation syndrome with aminoaciduria, iron overload, and lactic acidosis (GRACILE). Pediatr. Pathol. Mol. Med..

[108] Kotarsky H., Karikoski R., Mörgelin M., Marjavaara S., Bergman P., Zhang D.L., Smet J., van Coster R., Fellman V. (2010). Characterization of complex III deficiency and liver dysfunction in GRACILE syndrome caused by a BCS1L mutation. Mitochondrion.

[109] de Lonlay P., Valnot I., Barrientos A., Gorbatyuk M., Tzagoloff A., Taanman J.W., Benayoun E., Chrétien D., Kadhom N., Lombès A. (2001). A mutant mitochondrial respiratory chain assembly protein causes complex III deficiency in patients with tubulopathy, encephalopathy and liver failure. Nat. Genet..

[110] Serdaroğlu E., Takcı Ş., Kotarsky H., Çil O., Utine E., Yiğit Ş., Fellman V. (2016). A Turkish BCS1L mutation causes GRACILE-like disorder. Turk. J. Pediatr..

[111] Fellman V., Lemmelä S., Sajantila A., Pihko H., Järvelä I. (2008). Screening of BCS1L mutations in severe neonatal disorders suspicious for mitochondrial cause. J. Hum. Genet..

[112] Lee W.S., Sokol R.J. (2013). Mitochondrial hepatopathies: Advances in genetics, therapeutic approaches, and outcomes. J. Pediatr..

[113] Rajendran J., Tomašić N., Kotarsky H., Hansson E., Velagapudi V., Kallijärvi J., Fellman V. (2016). Effect of High-Carbohydrate Diet on Plasma Metabolome in Mice with Mitochondrial Respiratory Chain Complex III Deficiency. Int. J. Mol. Sci..

[114] Bardosi A., Creutzfeldt W., DiMauro S., Felgenhauer K., Friede R.L., Goebel H.H., Kohlschütter A., Mayer G., Rahlf G., Servidei S. (1987). Myo-, neuro-, gastrointestinal encephalopathy (MNGIE syndrome) due to partial deficiency of cytochrome-c-oxidase. A new mitochondrial multisystem disorder. Acta Neuropathol..

[115] Hirano M., Silvestri G., Blake D.M., Lombes A., Minetti C., Bonilla E., Hays A.P., Lovelace R.E., Butler I., Bertorini T.E. (1994). Mitochondrial neurogastrointestinal encephalomyopathy (MNGIE): Clinical, biochemical, and genetic features of an autosomal recessive mitochondrial disorder. Neurology.

[116] Nishino I., Spinazzola A., Hirano M. (1999). Thymidine phosphorylase gene mutations in MNGIE, a human mitochondrial disorder. Science.

[117] Nishino I., Spinazzola A., Papadimitriou A., Hammans S., Steiner I., Hahn C.D., Connolly A.M., Verloes A., Guimarães J., Maillard I. (2000). Mitochondrial neurogastrointestinal encephalomyopathy: An autosomal recessive disorder due to thymidine phosphorylase mutations. Ann. Neurol..

[118] Blondon H., Polivka M., Joly F., Flourie B., Mikol J., Messing B. (2005). Digestive smooth muscle mitochondrial myopathy in patients with mitochondrial-neuro-gastro-intestinal encephalomyopathy (MNGIE). Gastroenterol. Clin. Biol..

[119] Scarpelli M., Ricciardi G.K., Beltramello A., Zocca I., Calabria F., Russignan A., Zappini F., Cotelli M.S., Padovani A., Tomelleri G. (2013). The role of brain MRI in mitochondrial neurogastrointestinal encephalomyopathy. Neuroradiol. J..

[120] Hirano M., Nishigaki Y., Martí R. (2004). Mitochondrial neurogastrointestinal encephalomyopathy (MNGIE): A disease of two genomes. Neurologist.

[121] Garone C., Tadesse S., Hirano M. (2011). Clinical and genetic spectrum of mitochondrial neurogastrointestinal encephalomyopathy. Brain.

[122] Viscomi C., Zeviani M. (2017). MtDNA-maintenance defects: Syndromes and genes. J. Inherit. Metab. Dis..

[123] Filosto M., Tomelleri G., Tonin P., Scarpelli M., Vattemi G., Rizzuto N., Padovani A., Simonati A. (2007). Neuropathology of mitochondrial diseases. Biosci. Rep..

[124] Halter J., Schüpbach W., Casali C., Elhasid R., Fay K., Hammans S., Illa I., Kappeler L., Krähenbühl S., Lehmann T. (2011). Allogeneic hematopoietic SCT as treatment option for patients with mitochondrial neurogastrointestinal encephalomyopathy (MNGIE): A consensus conference proposal for a standardized approach. Bone Marrow Transplant..

[125] Wang J., Chen W., Wang F., Wu D., Qian J., Kang J., Li H., Ma E. (2015). Nutrition Therapy for Mitochondrial Neurogastrointestinal Encephalopathy with Homozygous Mutation of the TYMP Gene. Clin. Nutr. Res..

[126] Hirano M., Martí R., Casali C., Tadesse S., Uldrick T., Fine B., Escolar D.M., Valentino M.L., Nishino I., Hesdorffer C. (2006). Allogeneic stem cell transplantation corrects biochemical derangements in MNGIE. Neurology.

[127] Filosto M., Scarpelli M., Tonin P., Lucchini G., Pavan F., Santus F., Parini R., Donati M.A., Cotelli M.S., Vielmi V. (2012). Course and management of allogeneic stem cell transplantation in patients with mitochondrial neurogastrointestinal encephalomyopathy. J. Neurol..

[128] Halter J.P., Michael W., Schüpbach M., Mandel H., Casali C., Orchard K., Collin M., Valcarcel D., Rovelli A., Filosto M. (2015). Allogeneic haematopoietic stem cell transplantation for mitochondrial neurogastrointestinal encephalomyopathy. Brain.

[129] Lara M.C., Weiss B., Illa I., Madoz P., Massuet L., Andreu A.L., Valentino M.L., Anikster Y., Hirano M., Martí R. (2006). Infusion of platelets transiently reduces nucleoside overload in MNGIE. Neurology.

[130] Yavuz H., Ozel A., Christensen M., Christensen E., Schwartz M., Elmaci M., Vissing J. (2007). Treatment of mitochondrial neurogastrointestinal encephalomyopathy with dialysis. Arch. Neurol..

[131] Bax B.E., Bain M.D., Scarpelli M., Filosto M., Tonin P., Moran N. (2013). Clinical and biochemical improvements in a patient with MNGIE following enzyme replacement. Neurology.

[132] Röeben B., Marquetand J., Bender B., Billing H., Haack T.B., Sanchez-Albisua I., Schöls L., Blom H.J., Synofzik M. (2017). Hemodialysis in MNGIE transiently reduces serum and urine levels of thymidine and deoxyuridine, but not CSF levels and neurological function. Orphanet J. Rare Dis..

[133] De Giorgio R., Pironi L., Rinaldi R., Boschetti E., Caporali L., Capristo M., Casali C., Cenacchi G., Contin M., D’Angelo R. (2016). Liver transplantation for mitochondrial neurogastrointestinal encephalomyopathy. Ann. Neurol..

[134] Torres-Torronteras J., Cabrera-Pérez R., Barba I., Costa C., de Luna N., Andreu A.L., Barquinero J., Hirano M., Cámara Y., Martí R. (2016). Long-Term Restoration of Thymidine Phosphorylase Function and Nucleoside Homeostasis Using Hematopoietic Gene Therapy in a Murine Model of Mitochondrial Neurogastrointestinal Encephalomyopathy. Hum. Gene Ther..

[135] Barth P.G., Scholte H.R., Berden J.A., Van der Klei-Van Moorsel J.M., Luyt-Houwen I.E., Van ‘t Veer-Korthof E.T., Van der Harten J.J., Sobotka-Plojhar M.A. (1983). An X-linked mitochondrial disease affecting cardiac muscle, skeletal muscle and neutrophil leucocytes. J. Neurol. Sci.

[136] Lev D., Nissenkorn A., Leshinsky-Silver E., Sadeh M., Zeharia A., Garty B.Z., Blieden L., Barash V., Lerman-Sagie T. (2004). Clinical presentations of mitochondrial cardiomyopathies. Pediatr. Cardiol..

[137] Adès L.C., Gedeon A.K., Wilson M.J., Latham M., Partington M.W., Mulley J.C., Nelson J., Lui K., Sillence D.O. (1993). Barth syndrome: Clinical features and confirmation of gene localisation to distal Xq28. Am. J. Med. Genet..

[138] D’Adamo P., Fassone L., Gedeon A., Janssen E.A., Bione S., Bolhuis P.A., Barth P.G., Wilson M., Haan E., Orstavik K.H. (1997). The X-linked gene G4.5 is responsible for different infantile dilated cardiomyopathies. Am. J. Hum. Genet..

[139] Steward C.G., Newbury-Ecob R.A., Hastings R., Smithson S.F., Tsai-Goodman B., Quarrell O.W., Kulik W., Wanders R., Pennock M., Williams M. (2010). Barth syndrome: An X-linked cause of fetal cardiomyopathy and stillbirth. Prenat. Diagn..

[140] Clarke S.L., Bowron A., Gonzalez I.L., Groves S.J., Newbury-Ecob R., Clayton N., Martin R.P., Tsai-Goodman B., Garratt V., Ashworth M. (2013). Barth syndrome. Orphanet J. Rare Dis..

[141] Barth P.G., Valianpour F., Bowen V.M., Lam J., Duran M., Vaz F.M., Wanders R.J. (2004). X-linked cardioskeletal myopathy and neutropenia (Barth syndrome): An update. Am. J. Med. Genet. A.

[142] Brady A.N., Shehata B.M., Fernhoff P.M. (2006). X-linked fetal cardiomyopathy caused by a novel mutation in the TAZ gene. Prenat. Diagn..

[143] Rigaud C., Lebre A.S., Touraine R., Beaupain B., Ottolenghi C., Chabli A., Ansquer H., Ozsahin H., Di Filippo S., De Lonlay P. (2013). Natural history of Barth syndrome: A national cohort study of 22 patients. Orphanet J. Rare Dis..

[144] Bleyl S.B., Mumford B.R., Thompson V., Carey J.C., Pysher T.J., Chin T.K., Ward K. (1997). Neonatal, lethal noncompaction of the left ventricular myocardium is allelic with Barth syndrome. Am. J. Hum. Genet..

[145] Chin T.K., Perloff J.K., Williams R.G., Jue K., Mohrmann R. (1990). Isolated noncompaction of left ventricular myocardium. A study of eight cases. Circulation.

[146] Pignatelli R.H., McMahon C.J., Dreyer W.J., Denfield S.W., Price J., Belmont J.W., Craigen W.J., Wu J., El Said H., Bezold L.I. (2003). Clinical characterization of left ventricular noncompaction in children: A relatively common form of cardiomyopathy. Circulation.

[147] Spencer C.T., Bryant R.M., Day J., Gonzalez I.L., Colan S.D., Thompson W.R., Berthy J., Redfearn S.P., Byrne B.J. (2006). Cardiac and clinical phenotype in Barth syndrome. Pediatrics.

[148] Roberts A.E., Nixon C., Steward C.G., Gauvreau K., Maisenbacher M., Fletcher M., Geva J., Byrne B.J., Spencer C.T. (2012). The Barth Syndrome Registry: Distinguishing disease characteristics and growth data from a longitudinal study. Am. J. Med. Genet. A.

[149] Steward C.G., Groves S.J., Taylor C.T., Maisenbacher M.K., Versluys B., Newbury-Ecob R.A., Ozsahin H., Damin M.K., Bowen V.M., McCurdy K.R. (2019). Neutropenia in Barth syndrome: Characteristics, risks, and management. Curr. Opin. Hematol..

[150] Kelley R.I., Cheatham J.P., Clark B.J., Nigro M.A., Powell B.R., Sherwood G.W., Sladky J.T., Swisher W.P. (1991). X-linked dilated cardiomyopathy with neutropenia, growth retardation, and 3-methylglutaconic aciduria. J. Pediatr..

[151] Bolhuis P.A., Hensels G.W., Hulsebos T.J., Baas F., Barth P.G. (1991). Mapping of the locus for X-linked cardioskeletal myopathy with neutropenia and abnormal mitochondria (Barth syndrome) to Xq28. Am. J. Hum. Genet..

[152] Bione S., D’Adamo P., Maestrini E., Gedeon A.K., Bolhuis P.A., Toniolo D. (1996). A novel X-linked gene, G4.5. is responsible for Barth syndrome. Nat. Genet..

[153] Vreken P., Valianpour F., Nijtmans L.G., Grivell L.A., Plecko B., Wanders R.J., Barth P.G. (2000). Defective remodeling of cardiolipin and phosphatidylglycerol in Barth syndrome. Biochem. Biophys. Res. Commun..

[154] Gaspard G.J., McMaster C.R. (2015). Cardiolipin metabolism and its causal role in the etiology of the inherited cardiomyopathy Barth syndrome. Chem. Phys. Lipids.

[155] Houtkooper R.H., Turkenburg M., Poll-The B.T., Karall D., Pérez-Cerdá C., Morrone A., Malvagia S., Wanders R.J., Kulik W., Vaz F.M. (2009). The enigmatic role of tafazzin in cardiolipin metabolism. Biochim. Biophys. Acta.

[156] Valianpour F., Mitsakos V., Schlemmer D., Towbin J.A., Taylor J.M., Ekert P.G., Thorburn D.R., Munnich A., Wanders R.J., Barth P.G. (2005). Monolysocardiolipins accumulate in Barth syndrome but do not lead to enhanced apoptosis. J. Lipid Res..

[157] Kulik W., van Lenthe H., Stet F.S., Houtkooper R.H., Kemp H., Stone J.E., Steward C.G., Wanders R.J., Vaz F.M. (2008). Bloodspot assay using HPLC-tandem mass spectrometry for detection of Barth syndrome. Clin. Chem.

[158] Jefferies J.L. (2013). Barth syndrome. Am. J. Med. Genet. C Semin. Med. Genet..

[159] Meyerson C., Van Stavern G., McClelland C. (2015). Leber hereditary optic neuropathy: Current perspectives. Clin. Ophthalmol.

[160] Giraudet S., Lamirel C., Amati-Bonneau P., Reynier P., Bonneau D., Miléa D., Cochereau I. (2011). Never too old to harbour a young man’s disease?. Br. J. Ophthalmol..

[161] Barboni P., Savini G., Valentino M.L., La Morgia C., Bellusci C., De Negri A.M., Sadun F., Carta A., Carbonelli M., Sadun A.A. (2006). Leber’s hereditary optic neuropathy with childhood onset. Invest. Ophthalmol. Vis. Sci..

[162] Carelli V., Ross-Cisneros F.N., Sadun A.A. (2004). Mitochondrial dysfunction as a cause of optic neuropathies. Prog. Retin. Eye Res..

[163] Newman N.J. (1993). Leber’s hereditary optic neuropathy. New genetic considerations. Arch. Neurol..

[164] Chao de la Barca J.M., Simard G., Amati-Bonneau P., Safiedeen Z., Prunier-Mirebeau D., Chupin S., Gadras C., Tessier L., Gueguen N., Chevrollier A. (2016). The metabolomic signature of Leber’s hereditary optic neuropathy reveals endoplasmic reticulum stress. Brain.

[165] Jun A.S., Brown M.D., Wallace D.C. (1994). A mitochondrial DNA mutation at nucleotide pair 14459 of the NADH dehydrogenase subunit 6 gene associated with maternally inherited Leber hereditary optic neuropathy and dystonia. Proc. Natl. Acad. Sci. USA.

[166] Harding A.E., Sweeney M.G., Miller D.H., Mumford C.J., Kellar-Wood H., Menard D., McDonald W.I., Compston D.A. (1992). Occurrence of a multiple sclerosis-like illness in women who have a Leber’s hereditary optic neuropathy mitochondrial DNA mutation. Brain.

[167] Funalot B., Reynier P., Vighetto A., Ranoux D., Bonnefont J.P., Godinot C., Malthièry Y., Mas J.L. (2002). Leigh-like encephalopathy complicating Leber’s hereditary optic neuropathy. Ann. Neurol..

[168] Nikoskelainen E.K., Marttila R.J., Huoponen K., Juvonen V., Lamminen T., Sonninen P., Savontaus M.L. (1995). Leber’s “plus”: Neurological abnormalities in patients with Leber’s hereditary optic neuropathy. J. Neurol. Neurosurg. Psychiatry.

[169] Perez F., Anne O., Debruxelles S., Menegon P., Lambrecq V., Lacombe D., Martin-Negrier M.L., Brochet B., Goizet C. (2009). Leber’s optic neuropathy associated with disseminated white matter disease: A case report and review. Clin. Neurol. Neurosurg..

[170] Kellar-Wood H., Robertson N., Govan G.G., Compston D.A., Harding A.E. (1994). Leber’s hereditary optic neuropathy mitochondrial DNA mutations in multiple sclerosis. Ann. Neurol..

[171] Meire F.M., Van Coster R., Cochaux P., Obermaier-Kusser B., Candaele C., Martin J.J. (1995). Neurological disorders in members of families with Leber’s hereditary optic neuropathy (LHON) caused by different mitochondrial mutations. Ophthalmic Genet..

[172] Pfeffer G., Burke A., Yu-Wai-Man P., Compston D.A., Chinnery P.F. (2013). Clinical features of MS associated with Leber hereditary optic neuropathy mtDNA mutations. Neurology.

[173] Yu-Wai-Man P., Griffiths P.G., Brown D.T., Howell N., Turnbull D.M., Chinnery P.F. (2003). The epidemiology of Leber hereditary optic neuropathy in the North East of England. Am. J. Hum. Genet..

[174] Puomila A., Hämäläinen P., Kivioja S., Savontaus M.L., Koivumäki S., Huoponen K., Nikoskelainen E. (2007). Epidemiology and penetrance of Leber hereditary optic neuropathy in Finland. Eur. J. Hum. Genet..

[175] Mascialino B., Leinonen M., Meier T. (2012). Meta-analysis of the prevalence of Leber hereditary optic neuropathy mtDNA mutations in Europe. Eur. J. Ophthalmol..

[176] Finsterer J., Stöllberger C., Prainer C., Hochwarter A. (2004). Lone noncompaction in Leber’s hereditary optic neuropathy. Acta Cardiol..

[177] Finsterer J., Stöllberger C., Michaela J. (2002). Familial left ventricular hypertrabeculation in two blind brothers. Cardiovasc. Pathol..

[178] Ortiz R.G., Newman N.J., Manoukian S.V., Diesenhouse M.C., Lott M.T., Wallace D.C. (1992). Optic disk cupping and electrocardiographic abnormalities in an American pedigree with Leber’s hereditary optic neuropathy. Am. J. Ophthalmol..

[179] Finsterer J., Stollberger C., Gatterer E. (2018). Wolff-Parkinson-White syndrome and noncompaction in Leber’s hereditary optic neuropathy due to the variant m.3460G>A. J. Int. Med. Res..

[180] Nikoskelainen E.K., Savontaus M.L., Huoponen K., Antila K., Hartiala J. (1994). Pre-excitation syndrome in Leber’s hereditary optic neuropathy. Lancet.

[181] Mashima Y., Kigasawa K., Hasegawa H., Tani M., Oguchi Y. (1996). High incidence of pre-excitation syndrome in Japanese families with Leber’s hereditary optic neuropathy. Clin. Genet..

[182] Wallace D.C., Singh G., Lott M.T., Hodge J.A., Schurr T.G., Lezza A.M., Elsas L.J., Nikoskelainen E.K. (1988). Mitochondrial DNA mutation associated with Leber’s hereditary optic neuropathy. Science.

[183] Huoponen K., Vilkki J., Aula P., Nikoskelainen E.K., Savontaus M.L. (1991). A new mtDNA mutation associated with Leber hereditary optic neuroretinopathy. Am. J. Hum. Genet..

[184] Johns D.R., Neufeld M.J., Park R.D. (1992). An ND-6 mitochondrial DNA mutation associated with Leber hereditary optic neuropathy. Biochem. Biophys. Res. Commun..

[185] Riordan-Eva P., Harding A.E. (1995). Leber’s hereditary optic neuropathy: The clinical relevance of different mitochondrial DNA mutations. J. Med. Genet..

[186] Valentino M.L., Barboni P., Ghelli A., Bucchi L., Rengo C., Achilli A., Torroni A., Lugaresi A., Lodi R., Barbiroli B. (2004). The ND1 gene of complex I is a mutational hot spot for Leber’s hereditary optic neuropathy. Ann. Neurol..

[187] Valentino M.L., Avoni P., Barboni P., Pallotti F., Rengo C., Torroni A., Bellan M., Baruzzi A., Carelli V. (2002). Mitochondrial DNA nucleotide changes C14482G and C14482A in the ND6 gene are pathogenic for Leber’s hereditary optic neuropathy. Ann. Neurol..

[188] Chinnery P.F., Brown D.T., Andrews R.M., Singh-Kler R., Riordan-Eva P., Lindley J., Applegarth D.A., Turnbull D.M., Howell N. (2001). The mitochondrial ND6 gene is a hot spot for mutations that cause Leber’s hereditary optic neuropathy. Brain.

[189] Achilli A., Iommarini L., Olivieri A., Pala M., Hooshiar Kashani B., Reynier P., La Morgia C., Valentino M.L., Liguori R., Pizza F. (2012). Rare primary mitochondrial DNA mutations and probable synergistic variants in Leber’s hereditary optic neuropathy. PLoS ONE.

[190] Yu-Wai-Man P., Turnbull D.M., Chinnery P.F. (2002). Leber hereditary optic neuropathy. J. Med. Genet..

[191] Battisti C., Formichi P., Cardaioli E., Bianchi S., Mangiavacchi P., Tripodi S.A., Tosi P., Federico A. (2004). Cell response to oxidative stress induced apoptosis in patients with Leber’s hereditary optic neuropathy. J. Neurol. Neurosurg. Psychiatry.

[192] Giordano C., Montopoli M., Perli E., Orlandi M., Fantin M., Ross-Cisneros F.N., Caparrotta L., Martinuzzi A., Ragazzi E., Ghelli A. (2011). Oestrogens ameliorate mitochondrial dysfunction in Leber’s hereditary optic neuropathy. Brain.

[193] Sadun A.A., Carelli V., Salomao S.R., Berezovsky A., Quiros P.A., Sadun F., DeNegri A.M., Andrade R., Moraes M., Passos A. (2003). Extensive investigation of a large Brazilian pedigree of 11778/haplogroup J Leber hereditary optic neuropathy. Am. J. Ophthalmol..

[194] Kirkman M.A., Yu-Wai-Man P., Korsten A., Leonhardt M., Dimitriadis K., De Coo I.F., Klopstock T., Chinnery P.F. (2009). Gene-environment interactions in Leber hereditary optic neuropathy. Brain.

[195] Khan N.A., Govindaraj P., Soumittra N., Sharma S., Srilekha S., Ambika S., Vanniarajan A., Meena A.K., Uppin M.S., Sundaram C. (2017). Leber’s Hereditary Optic Neuropathy-Specific Mutation m.11778G>A Exists on Diverse Mitochondrial Haplogroups in India. Invest. Ophthalmol. Vis. Sci..

[196] Majander A., Bowman R., Poulton J., Antcliff R.J., Reddy M.A., Michaelides M., Webster A.R., Chinnery P.F., Votruba M., Moore A.T. (2017). Childhood-onset Leber hereditary optic neuropathy. Br. J. Ophthalmol..

[197] Johns D.R., Heher K.L., Miller N.R., Smith K.H. (1993). Leber’s hereditary optic neuropathy. Clinical manifestations of the 14484 mutation. Arch. Ophthalmol..

[198] Sadun A.A., Chicani C.F., Ross-Cisneros F.N., Barboni P., Thoolen M., Shrader W.D., Kubis K., Carelli V., Miller G. (2012). Effect of EPI-743 on the clinical course of the mitochondrial disease Leber hereditary optic neuropathy. Arch. Neurol..

[199] Klopstock T., Yu-Wai-Man P., Dimitriadis K., Rouleau J., Heck S., Bailie M., Atawan A., Chattopadhyay S., Schubert M., Garip A. (2011). A randomized placebo-controlled trial of idebenone in Leber’s hereditary optic neuropathy. Brain.

[200] Mashima Y., Hiida Y., Oguchi Y. (1992). Remission of Leber’s hereditary optic neuropathy with idebenone. Lancet.

[201] Mashima Y., Kigasawa K., Wakakura M., Oguchi Y. (2000). Do idebenone and vitamin therapy shorten the time to achieve visual recovery in Leber hereditary optic neuropathy?. J. Neuroophthalmol..

[202] Carelli V., La Morgia C., Valentino M.L., Rizzo G., Carbonelli M., De Negri A.M., Sadun F., Carta A., Guerriero S., Simonelli F. (2011). Idebenone treatment in Leber’s hereditary optic neuropathy. Brain.

[203] Pisano A., Preziuso C., Iommarini L., Perli E., Grazioli P., Campese A.F., Maresca A., Montopoli M., Masuelli L., Sadun A.A. (2015). Targeting estrogen receptor β as preventive therapeutic strategy for Leber’s hereditary optic neuropathy. Hum. Mol. Genet..

[204] Guy J., Qi X., Koilkonda R.D., Arguello T., Chou T.H., Ruggeri M., Porciatti V., Lewin A.S., Hauswirth W.W. (2009). Efficiency and safety of AAV-mediated gene delivery of the human ND4 complex I subunit in the mouse visual system. Invest. Ophthalmol. Vis. Sci..

[205] Ellouze S., Augustin S., Bouaita A., Bonnet C., Simonutti M., Forster V., Picaud S., Sahel J.A., Corral-Debrinski M. (2008). Optimized allotopic expression of the human mitochondrial ND4 prevents blindness in a rat model of mitochondrial dysfunction. Am. J. Hum. Genet..

[206] Yang S., Ma S.Q., Wan X., He H., Pei H., Zhao M.J., Chen C., Wang D.W., Dong X.Y., Yuan J.J. (2016). Long-term outcomes of gene therapy for the treatment of Leber’s hereditary optic neuropathy. EBioMedicine.

[207] Koilkonda R.D., Yu H., Chou T.H., Feuer W.J., Ruggeri M., Porciatti V., Tse D., Hauswirth W.W., Chiodo V., Boye S.L. (2014). Safety and effects of the vector for the Leber hereditary optic neuropathy gene therapy clinical trial. JAMA Ophthalmol..

[208] Guy J., Feuer W.J., Davis J.L., Porciatti V., Gonzalez P.J., Koilkonda R.D., Yuan H., Hauswirth W.W., Lam B.L. (2017). Gene Therapy for Leber Hereditary Optic Neuropathy: Low- and Medium-Dose Visual Results. Ophthalmology.

[209] Farruggia P., Di Cataldo A., Pinto R.M., Palmisani E., Macaluso A., Valvo L.L., Cantarini M.E., Tornesello A., Corti P., Fioredda F. (2016). Pearson Syndrome: A Retrospective Cohort Study from the Marrow Failure Study Group of A.I.E.O.P. (Associazione Italiana Emato-Oncologia Pediatrica). JIMD Rep..

[210] Rotig A., Bourgeron T., Chretien D., Rustin P., Munnich A. (1995). Spectrum of mitochondrial DNA rearrangements in the Pearson marrow-pancreas syndrome. Hum. Mol. Genet..

[211] Santorelli F.M., Barmada M.A., Pons R., Zhang L.L., DiMauro S. (1996). Leigh-type neuropathology in Pearson syndrome associated with impaired ATP production and a novel mtDNA deletion. Neurology.

[212] Lee H.F., Lee H.J., Chi C.S., Tsai C.R., Chang T.K., Wang C.J. (2007). The neurological evolution of Pearson syndrome: Case report and literature review. Eur. J. Paediatr. Neurol..

[213] McShane M.A., Hammans S.R., Sweeney M., Holt I.J., Beattie T.J., Brett E.M., Harding A.E. (1991). Pearson syndrome and mitochondrial encephalomyopathy in a patient with a deletion of mtDNA. Am. J. Hum. Genet..

[214] Mancuso M., Orsucci D., Angelini C., Bertini E., Carelli V., Comi G.P., Donati M.A., Federico A., Minetti C., Moggio M. (2015). Redefining phenotypes associated with mitochondrial DNA single deletion. J. Neurol..

[215] Crippa B.L., Leon E., Calhoun A., Lowichik A., Pasquali M., Longo N. (2015). Biochemical abnormalities in Pearson syndrome. Am. J. Med. Genet. A.

[216] Yamashita S., Nishino I., Nonaka I., Goto Y. (2008). Genotype and phenotype analyses in 136 patients with single large-scale mitochondrial DNA deletions. J. Hum. Genet..

[217] Puri A., Pradhan A., Chaudhary G., Singh V., Sethi R., Narain V.S. (2012). Symptomatic complete heart block leading to a diagnosis of Kearns-Sayre syndrome. Indian Heart J..

[218] Gobu P., Karthikeyan B., Prasath A., Santhosh S., Balachander J. (2011). Kearns Sayre Syndrome (KSS) - A Rare Cause For Cardiac Pacing. Indian Pacing Electrophysiol. J..

[219] van Beynum I., Morava E., Taher M., Rodenburg R.J., Karteszi J., Toth K., Szabados E. (2012). Cardiac arrest in kearns-sayre syndrome. JIMD Rep..

[220] Kabunga P., Lau A.K., Phan K., Puranik R., Liang C., Davis R.L., Sue C.M., Sy R.W. (2015). Systematic review of cardiac electrical disease in Kearns-Sayre syndrome and mitochondrial cytopathy. Int. J. Cardiol..

[221] Zeviani M., Servidei S., Gellera C., Bertini E., DiMauro S., DiDonato S. (1989). An autosomal dominant disorder with multiple deletions of mitochondrial DNA starting at the D-loop region. Nature.

[222] Kearns T.P., Sayre G.P. (1958). Retinitis pigmentosa, external ophthalmophegia, and complete heart block: Unusual syndrome with histologic study in one of two cases. AMA Arch. Ophthalmol..

[223] Akaike M., Kawai H., Yokoi K., Kunishige M., Mine H., Nishida Y., Saito S. (1997). Cardiac dysfunction in patients with chronic progressive external ophthalmoplegia. Clin. Cardiol..

[224] Aure K., Ogier de Baulny H., Laforet P., Jardel C., Eymard B., Lombes A. (2007). Chronic progressive ophthalmoplegia with large-scale mtDNA rearrangement: Can we predict progression?. Brain.

[225] Poulton J., Deadman M.E., Ramacharan S., Gardiner R.M. (1991). Germ-line deletions of mtDNA in mitochondrial myopathy. Am. J. Hum. Genet..

[226] Bernes S.M., Bacino C., Prezant T.R., Pearson M.A., Wood T.S., Fournier P., Fischel-Ghodsian N. (1993). Identical mitochondrial DNA deletion in mother with progressive external ophthalmoplegia and son with Pearson marrow-pancreas syndrome. J. Pediatr..

[227] Shanske S., Tang Y., Hirano M., Nishigaki Y., Tanji K., Bonilla E., Sue C., Krishna S., Carlo J.R., Willner J. (2002). Identical mitochondrial DNA deletion in a woman with ocular myopathy and in her son with pearson syndrome. Am. J. Hum. Genet..

[228] Remes A.M., Majamaa-Voltti K., Karppa M., Moilanen J.S., Uimonen S., Helander H., Rusanen H., Salmela P.I., Sorri M., Hassinen I.E. (2005). Prevalence of large-scale mitochondrial DNA deletions in an adult Finnish population. Neurology.

[229] Chinnery P.F., Johnson M.A., Wardell T.M., Singh-Kler R., Hayes C., Brown D.T., Taylor R.W., Bindoff L.A., Turnbull D.M. (2000). The epidemiology of pathogenic mitochondrial DNA mutations. Ann. Neurol..

[230] Kiechl S., Horvath R., Luoma P., Kiechl-Kohlendorfer U., Wallacher-Scholz B., Stucka R., Thaler C., Wanschitz J., Suomalainen A., Jaksch M. (2004). Two families with autosomal dominant progressive external ophthalmoplegia. J. Neurol. Neurosurg. Psychiatry.

[231] Harding A.E. (1981). Friedreich’s ataxia: A clinical and genetic study of 90 families with an analysis of early diagnostic criteria and intrafamilial clustering of clinical features. Brain.

[232] Durr A., Cossee M., Agid Y., Campuzano V., Mignard C., Penet C., Mandel J.L., Brice A., Koenig M. (1996). Clinical and genetic abnormalities in patients with Friedreich’s ataxia. N. Engl. J. Med..

[233] Koeppen A.H. (2011). Friedreich’s ataxia: Pathology, pathogenesis, and molecular genetics. J. Neurol. Sci..

[234] Jensen M.K., Bundgaard H. (2012). Cardiomyopathy in Friedreich ataxia: Exemplifying the challenges faced by cardiologists in the management of rare diseases. Circulation.

[235] Tsou A.Y., Paulsen E.K., Lagedrost S.J., Perlman S.L., Mathews K.D., Wilmot G.R., Ravina B., Koeppen A.H., Lynch D.R. (2011). Mortality in Friedreich ataxia. J. Neurol. Sci..

[236] Schultz J.C., Hilliard A.A., Cooper L.T., Rihal C.S. (2009). Diagnosis and treatment of viral myocarditis. Mayo Clin. Proc..

[237] Drinkard B.E., Keyser R.E., Paul S.M., Arena R., Plehn J.F., Yanovski J.A., Di Prospero N.A. (2010). Exercise capacity and idebenone intervention in children and adolescents with Friedreich ataxia. Arch. Phys. Med. Rehabil..

[238] Rustin P., von Kleist-Retzow J.C., Chantrel-Groussard K., Sidi D., Munnich A., Rotig A. (1999). Effect of idebenone on cardiomyopathy in Friedreich’s ataxia: A preliminary study. Lancet.

[239] Mariotti C., Solari A., Torta D., Marano L., Fiorentini C., Di Donato S. (2003). Idebenone treatment in Friedreich patients: One-year-long randomized placebo-controlled trial. Neurology.

[240] Lesnefsky E.J., Chen Q., Tandler B., Hoppel C.L. (2017). Mitochondrial Dysfunction and Myocardial Ischemia-Reperfusion: Implications for Novel Therapies. Annu. Rev. Pharmacol. Toxicol..

[241] Yellon D.M., Hausenloy D.J. (2007). Myocardial reperfusion injury. N. Engl. J. Med..

[242] Murphy E., Steenbergen C. (2008). Mechanisms underlying acute protection from cardiac ischemia-reperfusion injury. Physiol. Rev..

[243] Chen Q., Camara A.K., Stowe D.F., Hoppel C.L., Lesnefsky E.J. (2007). Modulation of electron transport protects cardiac mitochondria and decreases myocardial injury during ischemia and reperfusion. Am. J. Physiol. Cell Physiol..

[244] Lesnefsky E.J., Chen Q., Hoppel C.L. (2016). Mitochondrial Metabolism in Aging Heart. Circ. Res..

[245] Halestrap A.P., Clarke S.J., Javadov S.A. (2004). Mitochondrial permeability transition pore opening during myocardial reperfusion--a target for cardioprotection. Cardiovasc. Res..

[246] Kubli D.A., Gustafsson A.B. (2012). Mitochondria and mitophagy: The yin and yang of cell death control. Circ. Res..

[247] Kung G., Konstantinidis K., Kitsis R.N. (2011). Programmed necrosis, not apoptosis, in the heart. Circ. Res..

[248] Bugger H., Abel E.D. (2010). Mitochondria in the diabetic heart. Cardiovasc. Res..

[249] Rubler S., Dlugash J., Yuceoglu Y.Z., Kumral T., Branwood A.W., Grishman A. (1972). New type of cardiomyopathy associated with diabetic glomerulosclerosis. Am. J. Cardiol..

[250] Hamby R.I., Zoneraich S., Sherman L. (1974). Diabetic cardiomyopathy. JAMA.

[251] Regan T.J., Lyons M.M., Ahmed S.S., Levinson G.E., Oldewurtel H.A., Ahmad M.R., Haider B. (1977). Evidence for cardiomyopathy in familial diabetes mellitus. J. Clin. Investig..

[252] Bell D.S. (2003). Diabetic cardiomyopathy. Diabetes Care.

[253] Duncan J.G. (2011). Mitochondrial dysfunction in diabetic cardiomyopathy. Biochim. Biophys. Acta.

